# Evidence for phonon hardening in laser-excited gold using x-ray diffraction at a hard x-ray free electron laser

**DOI:** 10.1126/sciadv.adh5272

**Published:** 2024-02-09

**Authors:** Adrien Descamps, Benjamin K. Ofori-Okai, Oliviero Bistoni, Zhijiang Chen, Eric Cunningham, Luke B. Fletcher, Nicholas J. Hartley, Jerome B. Hastings, Dimitri Khaghani, Mianzhen Mo, Bob Nagler, Vanina Recoules, Ronald Redmer, Maximilian Schörner, Debbie G. Senesky, Peihao Sun, Hai-En Tsai, Thomas G. White, Siegfried H. Glenzer, Emma E. McBride

**Affiliations:** ^1^ SLAC National Accelerator Laboratory, 2575 Sand Hill Road, Menlo Park, CA 94025, USA.; ^2^ Aeronautics and Astronautics Department, Stanford University, 450 Serra Mall, Stanford, CA 94305, USA.; ^3^ School of Mathematics and Physics, Queen’s University Belfast, University Road, Belfast BT7 1NN, UK.; ^4^ PULSE Institute, SLAC National Accelerator Laboratory, 2575 Sand Hill Road, Menlo Park, CA 94025, USA.; ^5^ CEA/DAM DIF, F-91297 Arpajon Cedex, France.; ^6^ Université Paris-Saclay, CEA, Laboratoire Matière en Conditions Extrêmes, 91680 Bruyères-le-Châtel, France.; ^7^ Institut für Physik, Universität Rostock, Albert-Einstein-Straße 23, 18059 Rostock, Germany.; ^8^ University of Nevada, Reno, NV, 89557, USA.

## Abstract

Studies of laser-heated materials on femtosecond timescales have shown that the interatomic potential can be perturbed at sufficiently high laser intensities. For gold, it has been postulated to undergo a strong stiffening leading to an increase of the phonon energies, known as phonon hardening. Despite efforts to investigate this behavior, only measurements at low absorbed energy density have been performed, for which the interpretation of the experimental data remains ambiguous. By using in situ single-shot x-ray diffraction at a hard x-ray free-electron laser, the evolution of diffraction line intensities of laser-excited Au to a higher energy density provides evidence for phonon hardening.

## Teaser

X-ray diffraction at an x-ray free-electron laser reveals phonon hardening in laser-excited gold.

## INTRODUCTION

Over the past few decades, femtosecond optical-pump optical-probe measurements have enabled the investigation of ultrafast phenomena taking place in semiconductors (*1*, *2*) and metals (*3*–*6*) driven far from equilibrium. In these experiments, because of the small momentum of optical photons and the mass ratio between the electrons and the nuclei, the optical laser pulse transfers its energy primarily to the electronic subsystem, leaving the lattice initially unperturbed. This process can lead to exotic phenomena. For instance, simulations have predicted that under strong excitation, the interatomic potential of silicon softens to the point where transverse acoustic phonon modes become unstable and initiate a rapid disordering (*7*, *8*). Similarly, a softening of optical phonon modes caused by a solid-solid phase transition was observed in photo-excited bismuth using ultrafast x-ray diffraction measurements (*9*). In contrast, it has been postulated that the lattice response of metals upon strong optical excitation is fundamentally different. Density functional theory simulations performed on laser-excited gold (Au) predict that, when electrons near the Fermi surface are heated to a few electronvolts, while the lattice remains cold and at solid ambient density, a near-instantaneous stiffening of the interatomic potential occurs, caused by an increase of the strength of the metallic bonding (*8*, *10*). This hardening causes an increase of the phonon frequencies across the Brillouin zone and is referred to as phonon hardening. This behavior is expected to have an appreciable impact on the thermodynamic properties of Au under ultrafast intense irradiation as phonons contribute to intrinsic thermodynamic quantities such as constant-volume specific heat, entropy, and internal energy and hence would affect the melting behavior. In addition, phonon hardening is not a unique property of laser-excited Au, but is also expected to occur in other face cubic centered (fcc) metals such as Al, Cu, and Pt (*8*, *10*). Here, we focus on Au as a model system, allowing us to draw comparisons between previous experimental and theoretical studies.

The investigation of these phenomena requires measurements at the atomic scale with a time resolution on the order of phonon frequencies, which have recently been possible with the development of hard x-ray free electron lasers (XFELs) (*11*–*13*) and ultrafast electron diffraction facilities with megaelectronvolt energies (*14*–*16*). Ernstorfer *et al.* (*17*) have attempted to show evidence of phonon hardening by performing ultrafast electron diffraction measurements from 10 s of nanometer-thick Au foil excited using a femtosecond optical laser pulse. They used a two-temperature model (TTM) (*18*) to describe heating of the electronic and lattice subsystems after laser irradiation, combined with analysis of the intensity decay of the (2 2 0) diffraction line using the Debye-Waller theory. These observations were compared with simulations, which assumed an increase in the Debye temperature, Θ_D_, in laser-excited Au, an expected consequence of phonon hardening (*8*). However, recent modeling work using the ambient value of Θ_D_ (i.e., without assuming phonon hardening) (*10*) was able to reproduce the experimental data described in (*17*) As a result, the experimental observation of phonon hardening in laser-excited Au is still questioned and further investigations are required to provide evidence of this exotic behavior. More recently, also using ultrafast electron diffraction, Mo *et al.* (*16*) investigated the response of ultrafast heated nanometer-thick Au foils, but the electron temperature achieved was not sufficient to investigate phonon hardening (*10*).

Here, we describe the use of x-ray diffraction at a hard XFEL to measure the temporal evolution of the (1 1 1), (2 0 0), and (2 2 0) diffraction lines of laser-excited Au at an absorbed energy density of 6.4 ± 0.8 MJ/kg, an energy density more than two times larger than previously reported values (*17*) and for which the increase of the Θ_D_ is expected to be larger. The use of the ultra-bright x-ray pulses generated by the XFEL enables the collection of diffraction patterns on a single-shot basis with a temporal resolution of ~20 fs, an improvement in resolution by a factor of ~10 compared to previous measurements. In addition, the reciprocal space resolution achieved in this measurement is 1.6 × 10^−3^ Å^−1^, approximately two times better than electron diffraction measurements (*15*). As a result, we are sensitive to subtle changes of the diffraction peak positions, which allow us to only consider ambient solid density measurements, reducing the impact of complex hydrodynamic effects, and excluding density change effects, on the data interpretation. We find that the measured decay of the diffraction peak intensities is best explained by an increase of Θ_D_ and hence that our data provide evidence of the existence of phonon hardening in strongly excited Au.

## RESULTS

### Experimental method

**Fig. 1.**
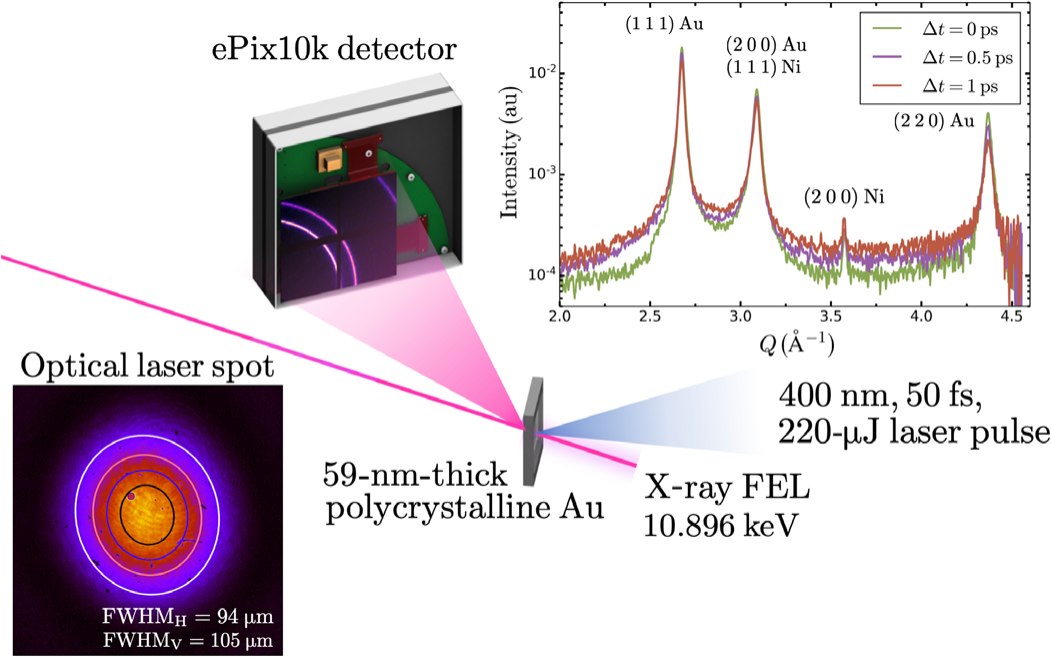
Schematic of the experimental setup used to measure the temporal evolution of the diffraction pattern from laser-heated, free standing Au foils at the LCLS. A transmission image of the nearly Gaussian transform-limited optical laser pulse is shown in the bottom left inset along with a set of contours corresponding to the best 2D Gaussian fit to the data. Azimuthally integrated diffraction patterns at different time delays are shown in the top right inset.

Experiments were conducted at the Matter in Extreme Conditions (MEC) endstation (*19*) of the Linac Coherent Light Source (LCLS) at SLAC National Accelerator Laboratory. A schematic of the experimental configuration is shown in Fig. 1. Free-standing 59-nm-thick Au foils (SciTech Ltd.) were irradiated using the MEC short-pulse laser system (*20*) frequency-doubled to 400 nm, providing ~222 ± 14 μJ in a 50-fs laser pulse. The spatially Gaussian optical laser pulse was focused to a spot size of ~100 μm–by–100 μm full width at half maximum (FWHM) at the target plane position. A transmission image of the laser spot at the target plane is shown in the bottom left inset of Fig. 1. Our films, excited to 6.4 ± 0.8 MJ/kg, were probed by the x-ray pulses at different time delays ranging from −2 to 3 ps with respect to the optical laser pulse in a single-shot basis. The timing between the optical laser pulse and the x-ray pulse was measured on a shot-to-shot basis using the time tool system available at the MEC endstation and was found to have an accuracy of 17 fs (see Materials and Methods).

Two-dimensional (2D) x-ray diffraction patterns were collected in transmission through the sample in a Debye-Scherrer geometry using a photon energy of 10.896 keV (λ = 1.138 Å). Examples of azimuthally integrated 1D diffraction patterns from laser-excited Au are shown in the top right inset of Fig. 1 for different time delays. The (1 1 1), (2 0 0), and (2 2 0) diffraction lines of Au are indicated and are indexed to give a lattice parameter of 4.071 ± 0.003 Å, in good agreement with the literature value (*21*). Weak diffraction lines from nickel (Ni), originating from the Ni mesh grid supporting the Au foils, can also be observed due to a low-intensity halo surrounding the focused x-ray beam at the target plane. This halo originates from the unfocused x-ray beam overfilling the beryllium compound refractive lenses (CRLs) used for focusing of the x-ray beam [the geometric aperture of the lenses is typically ~1 mm, and the unfocused beam is ~2 mm in diameter at the lenses position (*22*)]. The scattering signal from the halo contributes to the entire diffraction pattern in the same manner and is estimated to be ~20× smaller than the unheated Au signal and is hence negligible in this analysis (see Materials and Methods). The contribution from unexcited Au is found to be similarly small. As a result, the diffraction intensity from both unexcited Au and Ni is neglected in the analysis.

### Extraction of the Debye temperature

In the case of phonon hardening, the increase of the phonon energies upon excitation is driven by the excitation of the electronic system and is fundamentally different from the effect of density changes on the phonon dispersion relation. As a result, it is essential to ensure that the density of the system remains constant and equal to the ambient conditions value when investigating this exotic phenomenon. At our excitation conditions, the energy flow between the hot electrons and the lattice is fast enough to initiate a density change after 1 ps and drive a solid-liquid phase transition within 3 ps (see fig. S4). As a result, the measurements presented here were limited to a maximum time delay of 1 ps to satisfy the conditions for the investigation of phonon hardening. At later time delays, the diffraction line positions shift, indicating the onset of density changes (cf. Materials and Methods). Given this short temporal window, our improved time resolution of ~20 fs and the brightness of the x-ray pulse were essential to measure the intensity decay over the entire subpicosecond temporal window available on a single-shot basis. This is in contrast with the work of Ernstorfer *et al.* (*17*), which achieved a time resolution of ~400 fs and required data averaging. In addition, data, for which solid-liquid coexistence was observed, were included in their analysis, which violates the assumption of phonon hardening.

**Fig. 2.**
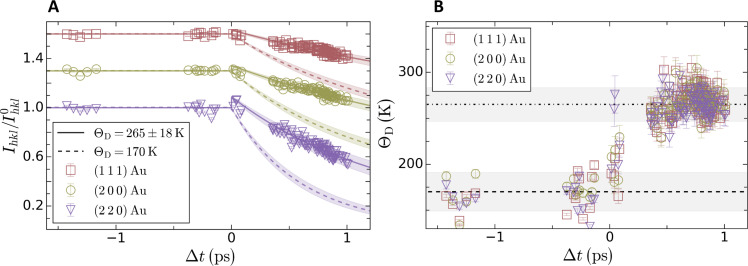
Extraction of the Debye temperature from the measured diffraction line intensity decays. (**A**) Temporal evolution of $I_{\mathrm{hkl}}/I_{\mathrm{hkl}}^{0}$ for the (1 1 1), (2 0 0), and (2 2 0) diffraction lines of Au shown with open squares, open circles, and open inverted triangles, respectively. For clarity, the data corresponding to the (2 0 0) and (1 1 1) diffraction peaks have been offset vertically by 0.3 and 0.6, respectively. The dashed curves correspond to the evolution simulated assuming that the Debye temperature remains constant to the ambient value. The solid curves were obtained using the Debye temperature extracted from the experimental data. The shaded areas correspond to the 1σ uncertainty. The uncertainty at positive delays is larger as it also considers the uncertainty on the deduced Debye temperature. (**B**) Temporal evolution of the Debye temperature measured using the (1 1 1), (2 0 0), and (2 2 0) diffraction line intensities. The horizontal dashed line indicates the literature value of the Debye temperature at ambient condition (*24*). The dashed-dotted line corresponds to the mean Debye temperature measured at positive time delays and was found to be Θ_D_ = 265 ± 18 *K*. The shaded area corresponds to the uncertainty of the Debye temperature measurement at the 1σ level. For negative time delays, the uncertainty corresponds to the deviation of the measured intensities from unity and is found to be 21 K.

Having established the temporal window for the measurement of phonon hardening, we normalize the diffraction lines of Au by the total number of counts recorded on the detector to account for fluctuation in the x-ray pulse energy. Each diffraction line is then integrated to give the intensity for each diffraction line, *I_hkl_* with *h*, *k*, and *l* corresponding to Miller indices. These intensities are finally normalized by the value without laser excitation, $I_{\mathrm{hkl}}^{0}$, and the result is shown by the open symbols in Fig. 2A for the (1 1 1), (2 0 0), and (2 2 0) diffraction lines of Au. More information on the extraction of the normalized intensity decay can be found in Materials and Methods.

The intensity decay of the (1 1 1), (2 0 0), and (2 2 0) diffraction lines over time exhibits the *Q*^2^ behavior expected from the Debye-Waller theory (*23*) and indicates an increase of the lattice temperature (see Materials and Methods). As a result, the decay of the diffraction lines of Au can be quantified by introducing the Debye-Waller factor, which requires knowledge of both the lattice temperature and Θ_D_. The first one is simulated using a TTM, described in details in Materials and Methods. At an absorbed energy density of 6.4 ± 0.8 MJ/kg, the maximum electron temperature reaches 3.5 ± 0.3 eV(41.1 ± 3 kK) and the lattice temperature is found to be 3.4 ± 0.3 kK(0.3 ± 0.03 eV) at the longest time delay of 1 ps. These values were obtained using parameters calculated with density functional theory simulations by Smirnov (*10*). However, the value of Θ_D_ at our excitation conditions is unknown. If its value is assumed to remain constant to the ambient value of 170 K (*24*), the simulated intensity decay (dashed curves in Fig. 2A) shows a clear deviation from our data. This suggests that the Debye temperature is changing at our excitation conditions. Here, unlike previous studies (*17*), no a priori assumption on the value of the Debye temperature is made. Instead, it is treated as a free parameter and is estimated by matching the simulated intensity decay to the experimentally measured value for each diffraction line and each time delay. More information on this procedure is found in Materials and Methods.

The extracted time-dependent Debye temperature is shown in Fig. 2B. The data show that the experimental diffraction intensities collected at an absorbed energy density of 6.4 ± 0.8 MJ/kg are consistent with an increase of the Debye temperature. Given the uncertainty on the deduced Debye temperature, the positive time delay data were found to be best described using a constant value of 265 ± 18 K as shown by the dashed-dotted horizontal line in Fig. 2B. This value is used to produce the solid lines in Fig. 2A. The reported uncertainty considers both the uncertainty on the timing between the optical laser pulse and the x-ray pulse, the uncertainty on the absorbed energy density, and the uncertainty on the measured diffraction line intensity (see Materials and Methods). The uncertainty at the 1σ level is shown by the shaded areas in Fig. 2.

## DISCUSSION

To simulate the evolution of the lattice temperature, *T*_l_, using a TTM, we need to know the electron-phonon coupling rate, *g*_ep_. However, there is no consensus on its value at our excitation conditions (*25*). To account for the influence of this parameter, we extract the Debye temperature using different electron-phonon coupling rates found in the literature: Smirnov (*10*), Holst *et al.* (*26*), Lin *et al.* (*27*), and Migdal *et al.* (*28*). The values obtained from (*26*, *27*) used in this analysis correspond to upper bounds on the temperature-dependent electron-phonon coupling rate, while the value obtained from Migdal *et al.* (*28*) corresponds to a lower bound. While the calculations from Smirnov (*10*), Holst *et al.* (*26*), and Lin *et al.* (*27*) are all based on the work by Allen (*29*), Smirnov uses the full spectral function, whereas Holst *et al.* (*26*) use its representation by the mass enhancement factor. Migdal *et al.* (*28*) use a slightly different theory, but more importantly, the density of states for Au is described using parabolic functions with the position of the electronic *d*-band kept fixed at an experimental value for all the electronic temperatures explored in their work. Note that for each model, the electron-phonon coupling rate and the electron heat capacity are calculated with the same electronic density of states.

**Fig. 3.**
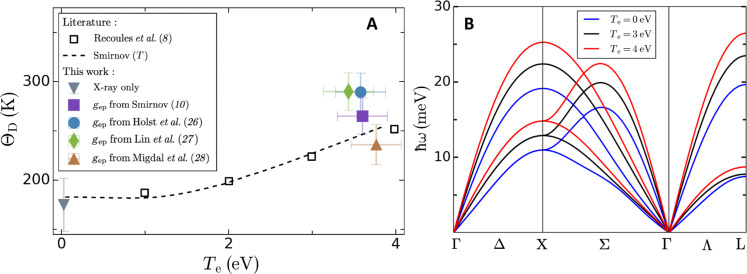
Comparison between the extracted Debye temperature and simulations in the event of phonon hardening. (**A**) Deduced Debye temperature for each electron-phonon coupling rate as a function of the electron temperature along with the theoretical predictions of phonon hardening from Recoules *et al.* (*8*) (open black squares) and Smirnov (*10*) (dashed line). The values obtained using the models from Smirnov (*10*), Holst *et al.* (*26*), Lin *et al.* (*27*), and Migdal *et al.* (*28*) are shown by the purple square, blue circle, brown triangle, and green diamond, respectively. The gray inverted triangle corresponds to the Debye temperature extracted from x-ray only measurements. For the experimental results, the electron temperature corresponds to the value obtained from the TTM simulations. The vertical error bars correspond to the 1σ-level uncertainty. (**B**) Calculated phonon dispersion curves of Au along high-symmetry paths in the first Brillouin zone showing the increase of the phonon energies characteristic of phonon hardening for a lattice at 0 K and electron temperatures comparable with our measurements. The blue curves correspond to the phonon dispersion of Au at zero electron temperature.

The results for different electron-phonon coupling rates are shown with different symbols in Fig. 3A. The Debye temperature calculated for each model considered in this work shows that an increase is necessary to explain our experimental data. Our measurements are compared with predictions from density functional theory (open squares and dashed line in Fig. 3A). The predicted Debye temperatures are found by matching the lattice heat capacity calculated within the Debye model with the one calculated from the phonon density of states obtained from first-principles calculations. The increase of the Debye temperature is a direct consequence of the increase of the phonon mode energies with increasing electron temperature and thus does not require the use of the TTM. To show this, we reproduce the work of Recoules *et al.* (*8*) and calculate the phonon dispersion of Au at 3- and 4-eV electron temperature (we achieved 3.5 ± 0.3 eV in this experiment) using the projector augmented wave method (*30*) as implemented in the ABINIT package (*31*–*33*). We use the Jollet-Torrent-Holzwarth (*34*) atomic dataset for Au within the local density approximation (*35*) and a plane-wave expansion up to 30-Ha cutoff. The Brillouin zone is sampled with a 32 × 32 × 32 *k*-points grid. Phonons are calculated on an 8 × 8 × 8 *q*-points grid. When increasing the electronic temperature, we include excited occupied states. We find that the energy of the phonon modes increases across the entire Brillouin zone as shown in Fig. 3B. This leads to a shift of the phonon density of states to higher energies and hence an increase of the Debye temperature. In Fig. 3A, the Debye temperatures shown with the open squares at 3 and 4 eV are calculated from the lattice heat capacity using the phonon density of states corresponding to the phonon dispersions in Fig. 3B.

From Fig. 3A, we observe that our measurements show an increase of Θ_D_ for all the values representative of the uncertainty on the electron-phonon coupling rate and thus provide further evidence for phonon hardening in laser-excited Au. We note that only the value from Lin *et al.* (*27*) was considered in the analysis from Ernstorfer *et al.* (*17*). Because of its high electron-phonon coupling rate, it predicts an upper bound on Θ_D_ as seen from Fig. 3A. However, the experimental data from Ernstorfer *et al.* (*17*) were also found to be in good agreement when using the values of the electron-phonon coupling rate and the electron heat capacity from Smirnov (*10*). For completeness, we also considered the opposite behavior corresponding to a sudden disappearance of bonding as observed in cubic diamond-structured semiconductors (*1*, *2*, *8*) and showed that this scenario does not reproduce the experimentally measured intensity decay of the diffraction peaks. The results of this analysis can be found in the Supplementary Materials.

In this study, we used in situ x-ray diffraction measurements at a hard XFEL to investigate phonon hardening in ultrafast laser-excited Au. With the much-improved time resolution of our measurement and the high brightness of the x-ray pulse, we provided additional evidence for phonon hardening at higher absorbed energy densities compared to previous studies (*17*). This work extends previous work by considering different values for the temperature-dependent electron-phonon coupling rate when calculating the lattice temperature and by showing that an increase of the Debye temperature is required to explain our experimental observations, for all coupling rates considered.

Here, we used a TTM to characterize the energy transfer between the electron subsystem and the lattice subsystem. While being widely used in the literature, this model assumes that all phonon branches in the system equilibrate instantaneously. To address this limitation, more sophisticated models such as the nonlinear model (NLM) proposed by Waldecker *et al.* (*36*) or the out-of-equilibrium dynamical model by Maldonado *et al.* (*37*) have been introduced. For this reason, we also considered the NLM, in addition to the TTM, to describe heating of the various phonon branches by the electron subsystem. However, the NLM requires knowledge of several additional coupling parameters compared to the TTM (phonon-phonon coupling rates for each phonon branch). Following the methodology outlined by Waldecker *et al.* (*36*), we performed an approximate calculation of these parameters. The simulation results indicate that the mean square atomic displacement aligns with the predictions of the TTM. Additional details can be found in the Supplementary Materials. Last, the model introduced by Maldonado *et al.* (*37*) surpasses the NLM by incorporating wave vector–dependent coupling parameters. While the calculation of these parameters at various electronic temperatures is in principle feasible with ab-initio calculations, this falls beyond the scope of this manuscript. For these reasons, we conclude that the TTM is the most suitable model available to describe heating of a Au lattice using ultrashort laser irradiation.

For the specific case of phonon hardening, recent developments at hard XFELs could provide an avenue to directly observe this behavior by measuring the increase of the phonon energies following laser excitation. This could be achieved using inelastic x-ray scattering with millielectronvolt resolution. This technique has been extensively used at synchrotron light sources (*38*) and has been recently fielded at XFELs (*39*–*41*) to take advantage of the exquisite temporal resolution required for ultrafast dynamics such as phonon hardening. We have demonstrated an energy resolution of 22 meV sufficient to resolve phonon modes near the edge of the Brillouin zone in ambient Au (*42*). In the presence of phonon hardening, the shift of the phonon energies to higher values is expected to be measurable by a shift of the inelastic components to larger energy transfers.

## MATERIALS AND METHODS

### Experimental details

The LCLS was operated in the hard x-ray self-seeding beam mode (*43*) at an incident x-ray photon energy of 10.896 keV with a nominal pulse duration of 50 fs and a bandwidth of 1 eV (Δ*E*/*E*). Using beryllium CRLs (Be CRLs) located 4 m upstream of the MEC vacuum chamber, the x-rays were focused on target to a spot size of ~20 μm by 20 μm FWHM.

The target consisted of 59 ± 2-nm-thick (measured along the x-ray propagation direction) polycrystalline Au foils grown by Scitech Precision Ltd. and deposited on top of a nickel (Ni) mesh grid from Goodfellow Cambridge Ltd. with wire diameter of 41 μm resulting in open squares of 340 μm in width, over which free-standing Au foils were suspended. The free-standing Au samples were irradiated using a 0° incidence, 750-mm focal length concave mirror operated at an angle of ~11° with respect to the incident x-ray beam. The absorption ratio of solid density Au at a wavelength of 400 nm is taken to be 0.47 ± 0.05 based on ex situ reflection and transmission measurements of our thin films and corresponds to the fraction of the incident optical laser energy deposited inside the film. Considering the area probed by the x-ray pulse, with these parameters, our samples were excited to an absorbed energy density of 6.4 ± 0.8 MJ/kg corresponding to an absorbed laser intensity of 1.5 ± 0.2 × 10^13^ W/cm^2^ and an absorbed laser fluence of 0.7 ± 0.1 J/cm^2^.

XRD was collected using an ePix10k detector (*44*) with 100 μm × 100 μm pixels. The sample-detector distance is ~130 mm, covering a 2θ range from 10.4° to 48.1°, corresponding to a momentum transfer range from *Q* = 1.0 to 4.5 Å^−1^, where $Q=\frac{4\pi }{\lambda }\text{sin}\theta$, with λ the x-ray photon wavelength.

### On-shot timing between the x-ray pulse and the optical laser pulse

#### Description of the time tool

**Fig. 4.**
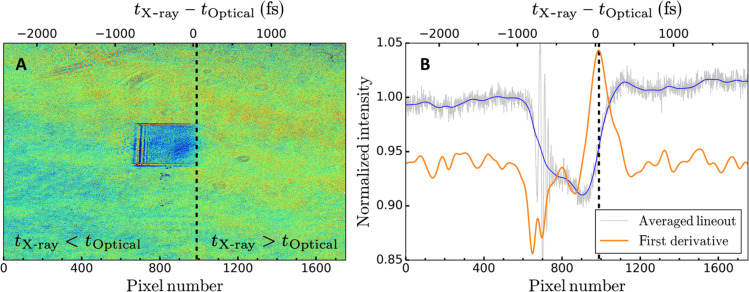
Shot-to-shot measurement of the time delay between the optical laser pulse and the x-ray pulse. (**A**) Background normalized image of the YAG window upstream of the vacuum chamber illuminated using the optical laser pulse. The window is angled, such that time is encoded in the horizontal axis. The vertical dashed line corresponds to the time at which the two pulses arrive simultaneously on the YAG window and is determined by the position of the maximum of the first derivative of the transient time tool signal as shown by the orange curve in (**B**).

Given the ultrafast nature of phonon hardening, the relative time of arrival between the optical laser pulse and the x-ray pulse needs to be known with high accuracy. For this, the MEC endstation uses the ultrafast excitation of a yttrium-aluminum-garnet (YAG) window after irradiation by an x-ray pulse to monitor the relative timing between the optical laser pulse and the x-rays and is referred to as the time tool. Here, a 100-μm-thick YAG window is positioned at normal incidence to the x-ray beam, 4 m upstream of the vacuum chamber. Upon irradiation by the x-ray pulse, the excitation causes the window to become opaque for optical radiation as the carrier density increases. A leakage of the optical laser pulse is then impinging on the YAG window at a 45° incidence angle such that the temporal information is encoded on one of the spatial axes. Typical images of the time tool are shown in Fig. 4A. The transmission decreases when the x-ray pulse is impinging on the YAG window first (blue area in Fig. 4A). By monitoring the position of the intensity recovery edge along the horizontal direction on a shot-to-shot basis, the time delay between the x-ray pulse and the optical laser pulse can be measured with ~20-fs accuracy. Here, the intensity edge is found from the first derivative of the transient time tool signal (orange solid line), and the edge position is defined as the pixel number corresponding to the maximum value as indicated by the vertical dashed line in Fig. 4 (A and B). When computing the first derivative, the raw signal is first smoothed using a Gaussian filter with a standard deviation of 40 pixels to remove high-frequency noise (blue curve in Fig. 4B).

**Fig. 5.**
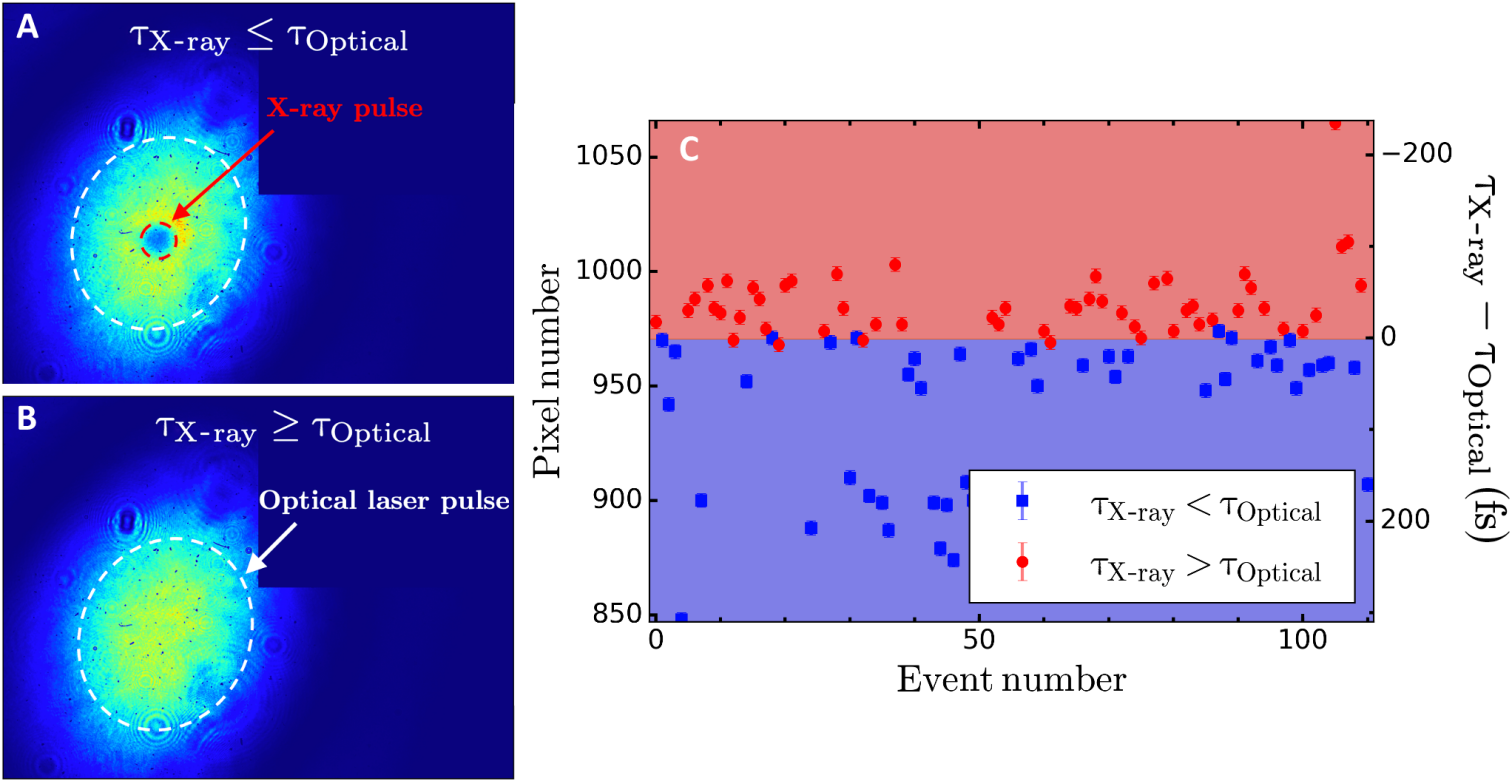
Determination of the zero time delay between the optical laser pulse and the x-ray pulse. Image of a 100-μm-thick YAG window at the target plane inside the vacuum chamber obtained after illumination by the optical laser when the x-ray pulse is impinging the window before the optical laser, τ_X-ray_ ≤ τ_Optical_, (**A**) and after the optical laser, τ_X-ray_ ≥ τ_Optical_, (**B**). The optical laser spot size is highlighted by the white dashed ellipse and the x-ray spot by the red dashed ellipse. The loss of intensity within the red dashed ellipse is a consequence of the transient change of the carriers density in the YAG window caused by the x-ray pulse, which, in turn, increases the absorption of optical light. (**C**) Pixel position found using the analysis shown Fig. 4 for each x-ray pulse (corresponding to each event number). The relative time of arrival between the x-ray pulse and the optical pulse is determined using the transmitted intensity through a 100-μm-thick YAG window positioned at the target plane for the same x-ray shots. Red corresponds to the optical pulse impinging the YAG window after the x-ray pulse, and blue corresponds to the optical laser arriving before the x-ray pulse. Here, τ_X-ray_ = τ_Optical_ corresponds to pixel number 971. We define this pixel position as zero time delay between the two pulses and refer to it as Δ*t* = 0 ps.

However, the time tool only provides the relative timing between the two pulses. For the analysis shown here, an absolute timing is necessary. This is obtained by correlating the timing measurement from the time tool with a secondary measurement performed on a 100-μm-thick YAG window positioned at the target plane inside the vacuum chamber. The latter can be used to determine the order of arrival between the x-ray pulse and the optical pulse on a shot-to-shot basis. We define τ_X-ray_ and τ_Optical_ as the time of arrival of the x-ray pulse and optical pulse at the target plane, respectively. By taking advantage of the inherent temporal jitter between the two pulses, one samples both τ_X-ray_ ≤ τ_Optical_ and τ_X-ray_ ≥ τ_Optical_ as shown in Fig. 5 (A and B). This allows the determination of the pixel position on the time tool images corresponding to τ_X-ray_ = τ_Optical_. This procedure is shown in Fig. 5C. Red circles correspond to the pixel position on the time tool images, for which τ_X-ray_ ≤ τ_Optical_. Blue squares correspond to the pixel position on the time tool images, for which τ_X-ray_ ≥ τ_Optical_. The pixel position corresponding to τ_X-ray_ = τ_Optical_ is found by finding the position of the line that best separates the two datasets. This is achieved using a support vector machine with a linear kernel. This line defines zero time delay between the two pulses and corresponds to Δ*t* = 0 ps. The pixel position corresponding to the edge on the time tool is then converted to absolute time, Δ*t*, using the time tool calibration (2.5 fs/pixel) and the pixel position corresponding to Δ*t* = 0 ps.

#### Estimation of the timing uncertainty between the optical laser pulse and the x-ray pulse

The uncertainty on Δ*t* is the combination of the relative timing uncertainty measured at the time tool and the uncertainty on the determination of the pixel position corresponding to Δ*t* = 0 ps. The relative timing uncertainty is estimated from the uncertainty on the determination of the edge position on the time tool images and is found to be 11 fs.

**Fig. 6.**
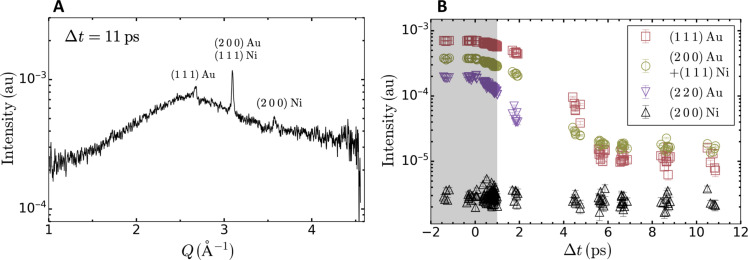
Estimation of the scattering signal from ambient conditions. (**A**) Azimuthally integrated diffraction pattern measured from laser-excited Au at 11-ps time delay. (**B**) Integrated intensity of the (1 1 1) Au (red squares), (2 0 0) Au + (1 1 1) Ni (green circles), (2 2 0) Au (purple inverted triangles), and (2 0 0) Ni (black triangles) diffraction lines. The latter peak cannot be distinguished from the background after 2-ps time delay. The shaded area between −2 and 1 ps corresponds to the time window considered for this analysis.

The uncertainty on the pixel position corresponding to Δ*t* = 0 ps is estimated from the classification boundary shown in Fig. 6. We notice that the two datasets are not perfectly separable. The uncertainty is taken to be the maximum distance between the outliers and the boundary and translates into a timing uncertainty of 13 fs. The uncertainties from both sources are lastly combined to give a timing uncertainty of 17 fs.

### Estimation of the intensity from the (1 1 1) diffraction line of Ni

Because of the lattice parameter of fcc Ni at ambient conditions, the (1 1 1) diffraction line of Ni was coincidentally overlapping with the (2 0 0) diffraction line of Au. Its contribution is estimated from x-ray diffraction data collected long after melting. The azimuthally integrated 1D x-ray diffraction pattern measured at 11 ps is shown in Fig. 6A. It shows a broad feature characteristic of a disordered state (liquid state) along with Au and Ni solid diffraction peaks. The solid peaks originate from the low-intensity halo surrounding the focused x-ray beam and, hence, far from the area irradiated by the optical laser. The diffraction lines are fitted using a pseudo-Voigt lineshape, allowing the extraction of the integrated intensity for each diffraction peak shown in Fig. 6B. One observes that the intensities of the (1 1 1) (red squares) and (2 0 0) (green circles) diffraction lines of Au and the intensity of (2 0 0) diffraction line of Ni (black triangles) reach a plateau above a time delay of 6 ps. These values are associated with the intensity scattered from the halo surrounding the focused x-ray beam. From the intensity of the (2 0 0) diffraction line of Ni, one concludes that this intensity is independent of the time delay. By comparing the intensity between early time delays (within the gray area in Fig. 6B) and long time delays (above 6 ps), we find that the summed contribution of the (2 0 0) diffraction line of ambient Au and the (1 1 1) diffraction line of ambient Ni contribute 20 times less to the intensity measured at ~3.08 Å^−1^ (green circles) and is thus neglected in the Debye-Waller analysis. For this reason, the green circles are labeled as “(2 0 0) Au.” Furthermore, the contribution from ambient Au to the diffraction intensity within the first picosecond is also neglected as the intensity of the (1 1 1) diffraction line above 6 ps is almost two orders of magnitude weaker. For this reason, the analysis assumes that the measured diffraction pattern is free of scattering from ambient materials.

### Extraction of the normalized intensity decay of the diffraction lines

**Fig. 7.**
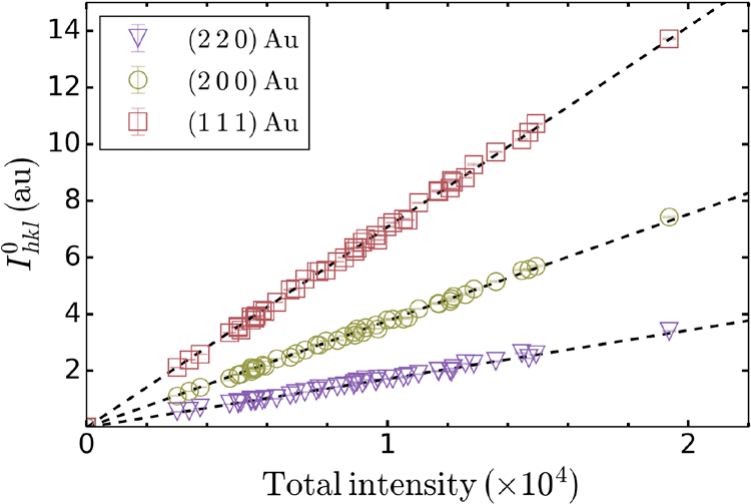
Correlation between the integrated diffraction peak intensity and the total intensity recorded on the detector. Intensity of the diffraction lines of Au as a function of the total intensity recorded on the 2D x-ray detector: (1 1 1) (red squares), (2 0 0) (green circles), and (2 2 0) (purple inverted triangles). All symbols correspond to x-ray only measurements. The intensity for each diffraction line, $I_{\mathrm{hkl}}^{0}$, was obtained by integrating the intensity found by fitting a pseudo-Voigt lineshape to each diffraction line. The dashed black lines correspond to the best linear fits to each dataset.

Each diffraction line of Au is fitted using a pseudo-Voigt lineshape from which the integrated intensity *I_hkl_* is calculated. To account for shot-to-shot fluctuations in the x-ray pulse energy, the integrated intensities are normalized by the total intensity recorded on the ePix10k x-ray detector. The values of the integrated intensity measured at ambient conditions without optical excitation, $I_{\mathrm{hkl}}^{0}$, are found to be linearly correlated with the total intensity recorded on the detector and shown by the black dashed lines in Fig. 7 for each diffraction line. Here, the x-ray pulse energy alone did not provide a good normalization as it is only measured at the exit of undulator, 100 s of meters upstream of the MEC endstation, and does not consider fluctuations in the beamlime transmission.

For all x-ray diffraction patterns recorded on laser-excited Au, the integrated diffraction intensity for each diffraction line is first calculated following the procedure described above and then normalized by the value without laser excitation. To calculate the latter one, the total intensity recorded on the x-ray detector corresponding to the laser-excited diffraction pattern and the linear fits shown in Fig. 7 are used. The quality of the normalization can be appreciated from the negative time delays in Fig. 2A as one expects these values to be unity.

### Determination of the temporal window

**Fig. 8.**
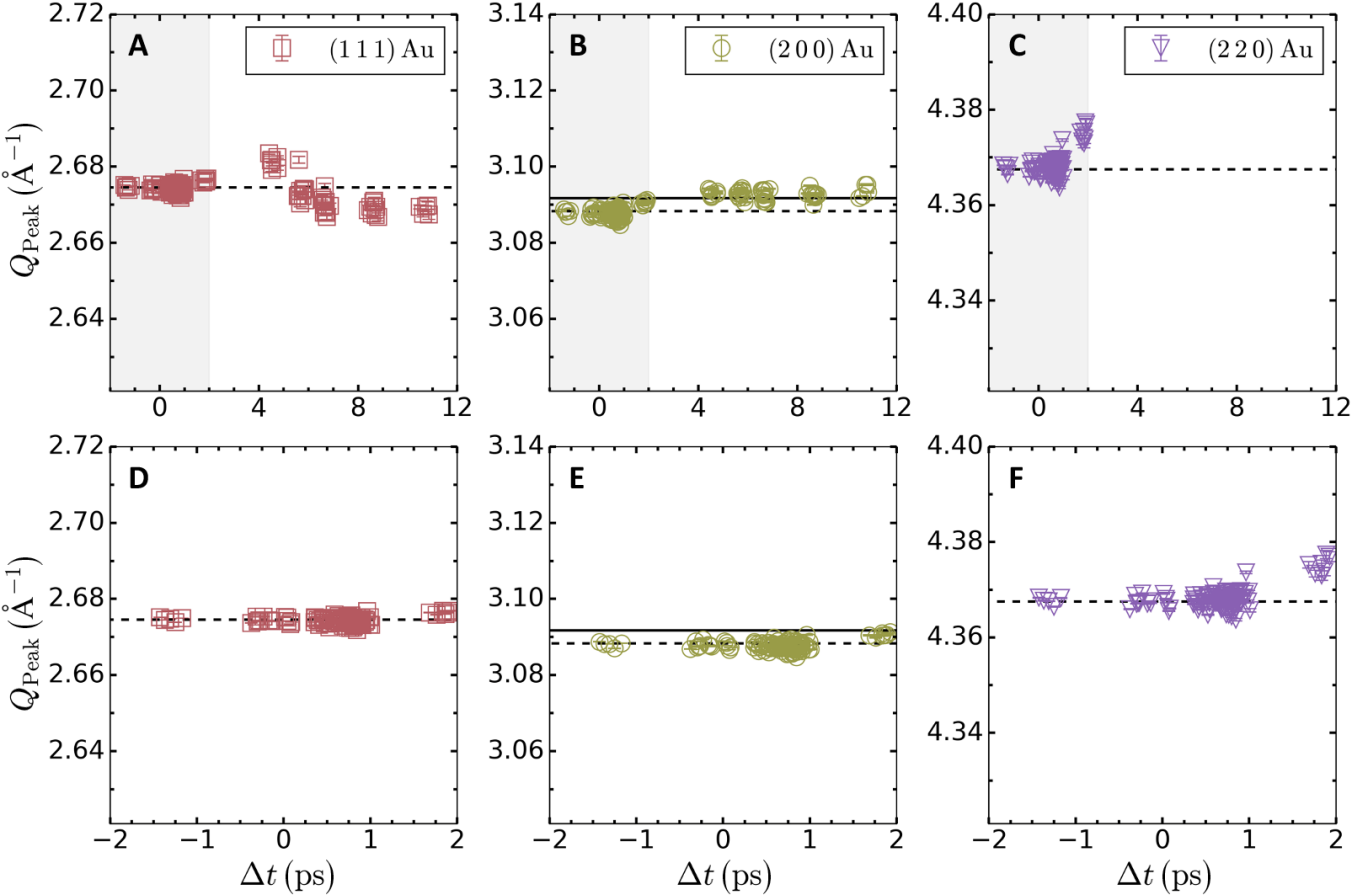
Temporal evolution of the diffraction peak position. Position, *Q*_Peak_, of the (1 1 1) (red squares), (2 0 0) (green circles), and (2 2 0) (purple inverted triangles) diffraction lines of Au as a function of the time delay. (**A** to **C**) The peak position between −2- and 12-ps time delays. (**D** to **F**) A zoom-in at early time delays between −2 and 2 ps, corresponding to the gray shaded area in (A) to (C). The black horizontal dashed lines in the bottom row correspond to the position of the diffraction lines of Au calculated using the lattice parameter found in the literature. The horizontal solid line in (E) corresponds to the position of the (1 1 1) diffraction line of Ni.

Because phonon hardening is predicted to happen for a Au lattice at ambient solid density, only data corresponding to these conditions are considered for the Debye-Waller analysis. From the position of the diffraction lines of Au shown in Fig. 8, we observe that the peak positions start shifting after 1-ps time delay. For this reason, only data collected between −2 and 1 ps time delays are used for the Debye temperature measurement.

### Justification for the Debye-Waller behavior of the diffraction line intensity decay

**Fig. 9.**
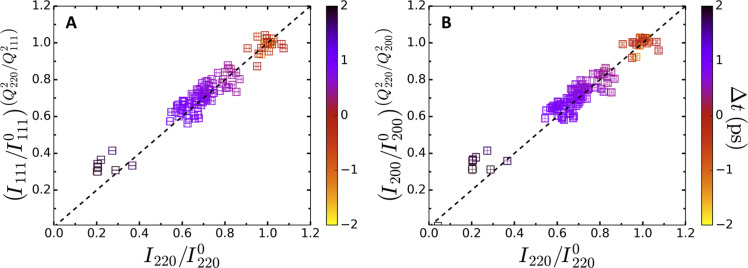
* Q*^2^ compensated-intensity of the diffraction peak intensities. Data compensated for the difference in the momentum transfer difference between the (2 2 0) and the (1 1 1) (**A**), as well as the (2 0 0) (**B**) diffraction lines. The black dashed line corresponds to the *Q*^2^ scaling expected from the Debye-Waller theory and is a line with unity slope. The different colors indicate different time delays between the x-ray pulse and the optical laser pulse.

The intensity decay of the diffraction peaks is given, within the Debye-Waller theory, by (*23*)

$$\frac{I_{\mathrm{hkl}}}{I_{\mathrm{hkl}}^{0}}=e^{-\frac{1}{3}\left[\langle u^{2}\rangle -\langle u_{0}^{2}\rangle \right].Q^{2}}$$ (1)

where *Q* is the momentum transfer and $\frac{1}{3}\langle u^{2}\rangle$ is the average mean square atomic displacement along each Cartesian directions. $\langle u_{0}^{2}\rangle$ is the mean square displacement at ambient conditions. The use of the average mean square atomic displacement is justified here as the crystallographic orientation of the samples is lost due to their polycrystalline nature. If the diffraction peak intensity decays observed in Fig. 2A are a consequence of the increase in the motion of atoms about their equilibrium position caused by an increase in temperature, then the decays are expected to follow a *Q*^2^ dependence. Because the mean square atomic displacement is independent of the momentum transfer, one can compensate for the difference in the momentum transfer between each diffraction line, such that the intensity of the (1 1 1) and (2 0 0) diffraction lines can be compared with the (2 2 0) diffraction line intensity. The result of this procedure is shown in Fig. 9. Here, the (2 2 0) diffraction line is used as the reference for the comparison because no diffraction line from Ni is expected at this momentum transfer. For each time delay, the data compensated for the difference in the momentum transfer are shown with open squares for the (1 1 1) diffraction line (Fig. 9A) and the (2 0 0) diffraction line (Fig. 9B). The black dashed line corresponds to the *Q*^2^ scaling expected from the Debye-Waller theory. We observe that the intensity decay of the (1 1 1) and (2 0 0) diffraction lines is consistent with the expected scaling, up to a time delay of 1 ps, thus justifying the use of the Debye-Waller theory to interpret our data.

### Debye temperature analysis methods

The evolution of the normalized diffraction line intensity is analyzed using the Debye-Waller theory such that the intensity decay is given by

$$\frac{I_{\mathrm{hkl}}}{I_{\mathrm{hkl}}^{0}}=\frac{e^{-2W\left(Q,T_{l}\right)}}{e^{-2W\left(Q,T_{l}^{0}\right)}}$$ (2)

with $T_{l}^{0}$ the ambient (room) lattice temperature. Within the framework of the Debye model (*23*, *45*), the Debye-Waller factor is given by

2W(Q,Tl)=3ℏ2Q2MkBΘD[14+(TlΘD)2∫0ΘDTlxex−1dx] (3)

where $x=\frac{\mathrm{\hbar \hbar}\omega }{k_{B}T_{l}}$, ω is the phonon frequency, *M* is the atomic mass, *k*_B_ is the Boltzmann constant, and *T*_l_ is the lattice temperature. Θ_D_ is the Debye temperature. The ambient temperature Debye-Waller factor, $2W\left(Q,T_{l}^{0}\right)$, is calculated using the literature value of Θ_D_ = 170 K (*24*).

To be used in Eqs. 2 and 3 requires both the Debye temperature and the lattice temperature. Here, the first one is treated as a free parameter that is calculated by matching the simulated and the experimental intensity decay of each diffraction peak. The lattice temperature is obtained from simulations of the energy flow between the hot electron population generated after laser irradiation and the lattice. In ultrafast excitation of metals using optical laser pulses, a TTM is commonly used and is given by the coupled partial differential Eq. 4 (*18*, *29*)

$$\begin{array}{c} C_{e}\left(T_{e}\right)\frac{\partial T_{e}}{\partial t}=-g_{\text{ep}}\left(T_{e}\right)\left(T_{e}-T_{l}\right)+S\left(t\right),\\ C_{l}\frac{\partial T_{l}}{\partial t}=+g_{\text{ep}}\left(T_{e}\right)\left(T_{e}-T_{l}\right) \end{array}$$ (4)

where *T*_e_ is the temperature of the electron population; *C*_l_ is the lattice heat capacity, taken to be equal to the Dulong-Petit limit (*46*); *C*_e_ is the electron temperature dependent electron heat capacity; *g*_ep_ is the electron temperature dependent electron-phonon coupling rate describing the energy transfer between the electronic subsystem and the lattice; and *S*(*t*) is a Gaussian source term accounting for the heating of the electron subsystem by the optical laser pulse.

In Eq. 4, both electronic and lattice heat conduction have been neglected as the associated time scales are much longer than the time scale of this measurement. The characteristic time of electronic heat conduction is estimated to be τ_e_ = *C*_e_*L*^2^/κ_e_ ∼ 10s of ps using the value for the electron heat diffusivity coefficient κ_e_ (*18*, *47*) and *L* = 50 nm. The lattice heat diffusion is also neglected since κ_l_ ≪ κ_e_. At an absorbed energy density of 6.4 ± 0.8 MJ/kg, the simulated electron temperature, simulated using the values from Smirnov (*10*), reaches a maximum of 3.5 ± 0.3 eV (41.1 ± 3 kK) and the simulated lattice temperature is found to be 3.4 ± 0.3 kK(0.3 ± 0.03 eV) at the longest time delay of 1 ps.

Given the laser parameters, the nonthermal electron population is expected to rapidly equilibrate through electron-electron collisions and is assumed to have fully thermalized on time scales shorter than electron-phonon interactions (*48*, *49*), thus justifying the use of a TTM in this work. It is further assumed that no temperature gradient is present in our target. The laser pulse energy is deposited uniformly throughout the sample thickness by energetic ballistic electrons as their effective absorption depth is ∼50 nm at our excitation conditions (*48*, *49*). At our excitation condition, these electrons travel at the Fermi velocity [~10^6^ m/s for Au (*50*)], thus reaching the back side of our target ~50 fs after laser irradiation.

To extract the Debye temperature, the temporal evolution of the electron and the lattice temperature are first simulated using a TTM with the source term corresponding to our laser excitation. It is taken to be Gaussian profile with a duration of 50-fs FWHM and normalized to match the incident optical laser pulse energy. Last, the values for the electron temperature–dependent electron heat capacity and the electron temperature–dependent electron-phonon coupling rate are taken from the values found in the literature for Au (*10*, *26*–*28*) and are shown in the fig. S1. The temporal evolution of the electron and lattice temperature calculated for each model are shown in the fig. S2.

From the temporal evolution of the lattice temperature, we invert Eqs. 2 and 3 to find the Debye temperature corresponding to each data point in Fig. 2A. This is numerically achieved using a least-square procedure that minimizes the distance between the simulated intensity decay and the measured intensity decay for a given diffraction line at a given time delay.

We note that, in Fig. 2A, the Debye temperature at negative time delays reflects the experimental uncertainty on the measured intensity decay of the diffraction lines. For these points, the right-hand side of Eq. 3 is unity and the Debye temperature should be identical to the literature value used to calculate the ambient temperature Debye-Waller factor. However, the left-hand side of Eq. 3 is not exactly unity due to experimental uncertainties and we express this as a varying Debye temperature to determine the precision of our measurement and to confirm that the increase measured at positive time delays is statistically relevant.

### Estimation of the uncertainty on the extracted Debye temperature

The uncertainty on the extracted Debye temperature is primarily due to the uncertainty on the measured intensity decay of the diffraction lines and the uncertainty on the lattice temperature. The latter is caused by timing uncertainties and uncertainties on the energy density absorbed by the sample.

#### Uncertainty on the absorbed energy density

The absorbed energy density is calculated from the fraction of the optical laser energy absorbed within the x-ray spot. The first source of uncertainty is the uncertainty on the incident optical laser pulse energy. The energy of the optical laser pulse corresponds to the maximum energy that could be used during the experiment. Because of the presence of spherical apertures along the beam path, upstream of the target, the laser spot at the target plane exhibits an Airy pattern for which the second Airy lobe overfills a single target aperture and contains ~14% of the laser pulse energy. This fraction can be sufficient to damage neighboring windows as the laser pulse energy is increased. For this reason, the maximum optical pulse energy that could be used was 222 ± 14 μJ. During the experiment, the incident laser pulse was imagined in transmission through the sample (bottom left inset in Fig. 1). The integrated number of counts on the transmission diagnostic was then calibrated using a powermeter. The reported uncertainty corresponds to the fluctuation in the incident laser energy extrapolated from the fluctuation in the integrated number of counts in the transmission diagnostic.

The second source of uncertainty is due to the misalignment between the x-ray pulse and the optical laser pulse, as well as the spatial jitter of the two beams. These are estimated from transmission images obtained using a 100-μm-thick YAG window at the target plane and corresponding to τ_X-ray_ ≤ τ_Optical_, as shown in Fig. 5A. Each pulse is fitted using a 2D Gaussian profile, from which the position of its center of mass is calculated. We found that the center of mass of the optical laser beam was slightly misaligned in the vertical axis by 12 μm. The energy contained with the 20-μm FHWM x-ray spot is then calculated to be 4.9 ± 0.3 μJ. The absorbed energy density is lastly calculated to be 6.4 ± 0.8 MJ/kg where the uncertainty considers the uncertainty on the incident optical pulse energy, the uncertainty on the absorption ratio of our thin films, and the spatial misalignment between the two pulses.

#### Uncertainty on the extracted Debye temperature

**Fig. 10.**
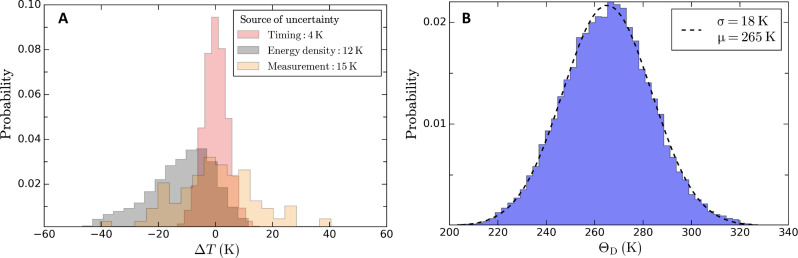
Estimation of the uncertainty on the extracted Debye temperature. (**A**) Distribution of the Debye temperature extracted from the experimental after propagation of the timing uncertainty only (red), the uncertainty on the absorbed energy density only (black), and the uncertainty on the intensity decay of the diffraction lines measured experimentally only (orange). The distributions are centered around the mean value of the extracted Debye temperature. (**B**) Distribution of the Debye temperature obtained after propagating the uncertainties from the three sources shown in (A). The distribution is fitted to a normal distribution and the result is shown with the black dashed line. The results are obtained using the TTM parameters provided by Smirnov.

To quantify the contribution from each source of uncertainty, we extract the Debye temperature by considering only one source of uncertainty at a time. The results obtained with the model from Smirnov are summarized in Fig. 10A. The uncertainty on the timing, the absorbed energy density, and the measured intensity decay of the diffraction lines altogether are estimated using a Monte Carlo error propagation procedure. The results of this procedure are shown in Fig. 10B. The same analysis was performed for the other models to extract the mean Debye temperature and the corresponding uncertainty.

## Supplementary Materials

### This PDF file includes:

Supplementary Text Figs. S1 to S8 References
