# Supplementary material for: Evidence for phonon hardening in laser-excited gold using x-ray diffraction at a hard x-ray free electron laser
Source: Sci Adv. 2024 Feb 9;10(6):eadh5272. doi: 10.1126/sciadv.adh5272 (PMC10857355; doi:10.1126/sciadv.adh5272)
Supplement: Supplementary file 1 — Supplementary Text Figs. S1 to S8 References [file sciadv.adh5272_sm.pdf]

Science Advances
AAAS

Supplementary Materials for

Evidence for phonon hardening in laser-excited gold using x-ray diffraction at a hard x-ray free electron laser

Adrien Descamps *et al.*

Corresponding author: Adrien Descamps, a.descamps@qub.ac.uk; Emma E. McBride, e.mcbride@qub.ac.uk

Sci. Adv. **10**, eadh5272 (2024)
DOI: 10.1126/sciadv.adh5272

This PDF file includes:

Supplementary Text
Figs. S1 to S8
References

Supplementary Text

Comparison of the electron heat capacity and electron phonon coupling rates found in the literature

To determine the time evolution of the electron and lattice temperatures, we use a two-temperature model (TTM) with an electron temperature dependent electronic heat capacity and electron-phonon coupling rate as shown in Fig. S1. During the integration of the coupled equations shown in Eq. 4 in the main manuscript, the electron heat capacity and electron-phonon coupling rate are calculated at the current electron temperature. For the integration a time step of 0.1 fs is used. We note that Smirnov (10) does not provide the electron heat capacity for gold. As a result, we used the electron heat capacity from Holst *et al.* (26) when solving the TTM with the electron-phonon coupling rate from Smirnov.

Extraction of the Debye temperature with different electron-phonon coupling rates

As mentioned in the main manuscript, computing the electron-phonon coupling rate from first principles simulations is computationally expensive and assumptions are often used. This leads to different values for the electron temperature-dependent electron-phonon coupling rates depending on the approximations made. To reflect the uncertainty on g_{ep}, we have considered the values from Smirnov (10), Holst *et al.* (26), Lin *et al.* (27), and Migdal *et al.* (28) with the last two values corresponding to upper and lower bounds respectively. Since the Two-Temperature Model simulations depend on the electron-phonon coupling rates, the different models give different conditions for the electron and lattice temperatures simulated at an absorbed energy density of 6.4 ± 0.8 MJ/kg, as shown in Fig. S2. The larger electron temperature observed using the values from Migdal *et al.* is a consequence of the smaller electron heat capacity simulated in their work. The simulated electron temperature within the first picosecond was found to be $3.5 \pm 0.3\ eV$ for Holst *et al.*, $3.7 \pm 0.3\ eV$ for Migdal *et al.*, and $3.4 \pm 0.3\ eV$ for Lin *et al.* At 1 ps, the lattice temperatures are respectively $4.1 \pm 0.4\ kK$ (Holst *et al.*), $2.7 \pm 0.4\ kK$ (Migdal *et al.*), and $4.1 \pm 0.4\ kK$ (Lin *et al.*). We note than despite using different parameters, the lattice temperature simulated using the models from Holst *et al.* and Lin *et al.* overlap in Fig. S2.

The different values of T_l lead to different values of the Debye temperature extracted from the experimental decay of the (1 1 1), (2 0 0), and (2 2 0) diffraction lines, as shown in Fig. S3 (A-C). The dash-dotted lines correspond to the mean values of the Debye temperature found for each electron-phonon coupling rate and are reported in Fig. 3 of the main manuscript. The corresponding simulated intensity decay of the diffraction lines is shown with the solid-colored lines in Fig. S3 (D-F).

Onset of melting from X-ray diffraction

The diffraction patterns collected without optical laser excitation, at 1 ps and 3 ps time delays are shown in Fig. S4 in green, purple, and gray respectively. At the excitation conditions used for this experiment, we observe the emergence of a broad scattering signal at ~ 3 ps. This signal is attributed to the onset of melting as it is centered around the (111) diffraction line of Au, which is expected for a *fcc* crystalline structure undergoing a solid-liquid phase transition.

Consideration of anharmonic effects on the lattice heat capacity

In our work, we have used the lattice heat capacity calculated within the harmonic approximation which only considers the second derivative of the interatomic potential when calculating the phonon properties of the system. We show in Fig. S5. the calculation of the lattice heat capacity from the phonon density of states in the harmonic approximation at several electron temperatures (HA lines in Fig. S5.). For a lattice temperature of 300 K, the heat capacity in the harmonic approximation is 4% lower than the Dulong-Petit limit (45). This error is assumed to be negligible compared to the other source of uncertainties when calculating the lattice temperature (the uncertainty on absorbed energy density is 12%) and completely vanishes as the lattice temperature increases above room temperature. For these reasons, we replace the lattice heat capacity by the Dulong-Petit limit.

However, as the lattice temperature increases, the harmonic approximation might break down due to phonon-phonon interaction. To ensure anharmonicity does not play a significant role in this experiment, we have investigated its effect using classical Molecular Dynamics simulation. In MD, calculations of the thermodynamics properties account for terms beyond the second derivative of the interatomic potential and are hence sensitive to anharmonic effects. In comparison, DFPT calculations are often limited to the harmonic approximation for which no interaction between phonons is considered.

To calculate the heat capacity beyond the harmonic approximation, we ran MD simulations in the NVT ensemble using the interatomic potential from G.E. Norman *et al.* (51). This potential has been constructed to reproduce the phonon properties calculated from DFT simulations for Au at different electron temperatures and thus includes the effect of phonon hardening. For the simulations, a 20x20x20 FCC lattice of Au, with a lattice constant of 4.16 Å, is first equilibrated for 10,000 timesteps and the total energy of the system (kinetic energy + potential energy) is then computed for 10,000 timesteps. This procedure is repeated for temperatures ranging from 50 K to 2500 K with 50 K increment. The heat capacity at constant volume is then calculated from the rate of change of the total energy with respect to the temperature.

$$\frac{\mathrm{C_V}}{\mathrm{k_B}} = \frac{1}{\mathrm{N}}\frac{\partial \mathrm{E}}{\partial \mathrm{T}}$$

Where T is the lattice temperature, N is the number of particles in the simulation box, E is the total energy of system.

The results are shown in Fig. S5 using the embedded atom model potential from G.E. Norman *et al.* (51) for an electron temperature of 3 eV (close to our experimental conditions). We observe that the heat capacity calculated with MD is in good agreement with the Dulong-Petit limit

across the temperature range of our experiment and thus suggests that anharmonic effects are not playing an important at our conditions. We note that the heat capacity calculated from MD at low temperature disagrees with the harmonic approximation because MD simulations are classical and do not consider quantization of phonons.

Comparison of the Two-Temperature Model with the Non-Linear Model

In this work, we have used a TTM to model the energy exchange between the electron subsystem and the lattice subsystem. This model assumes an instantaneous equilibration of the different phonon branches contributing to the lattice subsystem. To relax this assumption, we considered the non-linear model (NLM) described in Waldecker *et al.* (36) and applied it for Au. The main difference with a TTM is that each phonon branches is treated explicitly with its own electron-phonon coupling rate and each phonon branch is allowed to exchange energy with other branches through a phonon-phonon coupling rate. The equations governing the NLM are summarized below (36):

$$\mathrm{C_e}\frac{\partial \mathrm{T_e}}{\partial \mathrm{t}} = -\sum_{\mathrm{i}=1}^{\mathrm{N}} \mathrm{G_{ep,i}}\left(\mathrm{T_e} - \mathrm{T_{p,i}}\right) + \mathrm{S(t)}$$

$$\mathrm{C_{p,i}}\frac{\partial \mathrm{T_{p,i}}}{\partial \mathrm{t}} = \mathrm{G_{ep,i}}\left(\mathrm{T_e} - \mathrm{T_{p,i}}\right) + \sum_{\mathrm{j}\neq \mathrm{i}} \mathrm{G_{pp,ij}}\left(\mathrm{T_{p,j}} - \mathrm{T_{p,i}}\right)$$

Where N is the number of phonon branches in the system (3 for Au), $G_{ep,i}$ is the electron phonon coupling rate with the phonon branch i, $T_{p,i}$ is the temperature of the phonon branch i, $C_{p,i}$ is the heat capacity of the phonon branch i, and $G_{pp,ij}$ is the phonon-phonon coupling rate between phonon branches i and j.

The mode-dependent phonon heat capacities and electron-phonon coupling rates are calculated following the description in Waldecker *et al.* (36). The phonon heat capacities for each branch are calculated from the contribution of each phonon branch to the total phonon density of states, $F(\omega)$. The phonon density of states is calculated from the interatomic force constants obtained using the ABINIT package and by integrating over the Brillouin zone using a 50x50x50 q-point grid and a smearing value of 0.03 THz. The results for each phonon branch are shown in Fig. S6 (A). The mode-dependent electron-phonon coupling rates are calculated from the contribution of each phonon branch to the total Eliashberg spectral function, $\alpha^2F(\omega)$. For this, we followed the approximation used by Waldecker *et al.* which consists of dividing the total Eliashberg spectral function by the total phonon density of states to compute α^2. The mode-resolved Eliashberg spectral function is then computed by multiplying each partial phonon density of states by the calculated value of α^2.The results are shown in Fig. S6 (B).

Like Waldecker *et al.*, the phonon-phonon coupling rate is assumed to be the same for all the phonon branches, $\boldsymbol{G_{pp}}$. Since we did not find any experimental measurement of this term for laser-excited, we assume that this term is on the same order of magnitude as the electron-phonon coupling rate, taken to be $\mathbf{10^{17}}\ \boldsymbol{W.m^{-3}.K^{-1}}$. This was shown to be true for Al (52). In the limit

of an infinite $\boldsymbol{G_{pp}}$, all the phonon branches equilibrate instantaneously and the NLM reduces to the TTM.

To compare the TTM and the NLM, we simulate the temperature evolution of the electron and lattice subsystems and calculate the mean square displacement for each model. The results are shown in the Fig. S7.

From Fig. S7 (B), we observe that the LA branch is heated more rapidly that the transverse branches. This is due to the higher electron-phonon coupling rate with the LA mode which represents the tendency of the electron subsystem to couple preferentially to high energy phonons. We note that these high energy phonons would be found at high wavevectors $\boldsymbol{q}$, close to the edge of the Brillouin zone.

In addition, we observe in Fig. S7 (C) that the mean square displacements predicted by each model are consistent with each other. Even though the NLM gives a more sophisticated picture of the energy transfer between the electron subsystem and the lattice subsystem, this model requires a phonon-phonon coupling rate which is difficult to calculate using current DFT capabilities. Since this parameter is much less constrained (both experimentally and computationally) than the electron-phonon coupling rate, the prediction of the NLM seems more uncertain than the prediction of the TTM. For this reason, we conclude that the TTM is the best model currently accessible to describe the energy relaxation for our experiment.

Expected intensity decay in case of a sudden disappearance of bonding

In this scenario, we consider that the interatomic potential instantaneously disappears after irradiation by the optical laser pulse. Following the procedure highlighted by Wang *et al.* (53), we simulated the intensity decay of the diffraction peaks of gold assuming that each atom follows a ballistic trajectory given by its velocity vector immediately before laser irradiation. First, we performed MD simulations of Au at 300 K. For the simulation we used a computation cell of 10x10x10 *fcc* unit cells with 4 atoms per unit cell with periodic boundary conditions. The simulation was first equilibrated for 50,000 steps and then 2,000 configurations were sampled to obtain the positions and velocities of the atoms.
Using these velocities, we constructed the atom trajectories over 1 ps (timescale of our measurement) assuming they follow a ballistic motion. We calculated the intensity decay at each time step for the (1 1 1), (2 0 0), and (2 2 0) diffraction peaks observed experimentally and for each configuration by computing the static structure factor, S(Q), directly from the atomic positions using the *dynasor* package (54). The results, averaged over all configurations, are shown with the black dashed lines in Fig. S8. We also show the evolution calculated using the thermal velocity of gold atoms at 300 K, solid black lines. From the MD simulations, we obtain $v_{th} \sim 1.8\ \text{Å}/ps$ for Au at 300 K.

Assuming a sudden disappearance of bonding, we find that the intensity decay of the diffraction peak intensity is substantially faster and does not explain our experimental observations. From this analysis, we conclude that this hypothesis can be ruled out.

The work from Wang *et al.* was performed on InSb, a semiconductor with cubic diamond structure. The response of semiconductors with the cubic diamond structure (Si, Ge, InSb) to intense femtosecond excitation has been extensively studied and was found to be fundamentally different from the response of metals. In the case of diamond structure semiconductors, the laser pulse excites carriers from the bonding valence band to the anti-bonding conduction band, which destabilizes the system. As the laser excitation increases, the solid phase becomes more unstable which initiates a rapid disordering of the system (1, 8, 55, 56). In comparison, this transition from bonding and anti-bonding bands doesn't happen for Au and explains the different responses to intense ultrafast excitation.

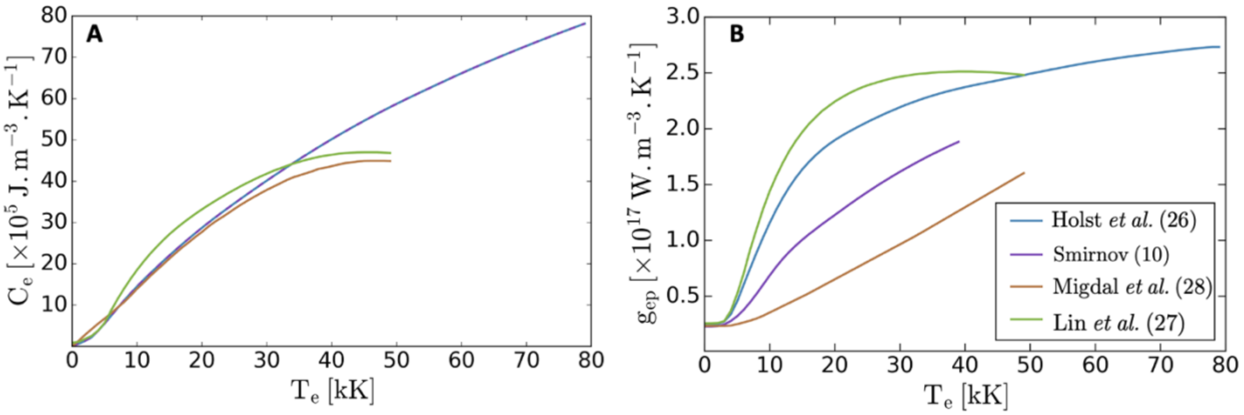

Fig. S1. Electron heat capacity (A) and electron-phonon coupling rate (B) as a function of the electron temperature. The different values used in the analysis are shown with different colors: Smirnov (purple), Holst *et al.* (blue), Lin *et al.* (green), and Migdal *et al.* (brown).

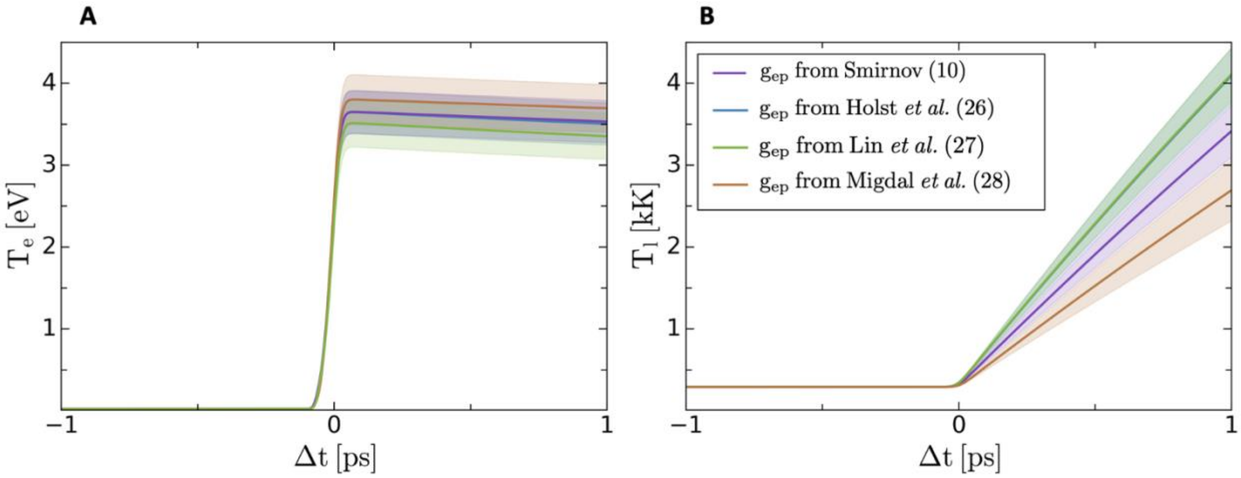

Fig. S2. Temporal evolution of the electron temperature (A) and the lattice temperature (B). The evolution is simulated using a TTM with the electron temperature dependent electron-phonon coupling rate from Smirnov (purple), Holst *et al.* (blue), Lin *et al.* (green), and Migdal *et al.* (brown). The simulations were all performed at an absorbed energy density of 6.4 ± 0.8

MJ/kg. The shaded areas correspond to the 1σ-level uncertainty region obtained from the propagation of the uncertainty on the absorbed energy density.

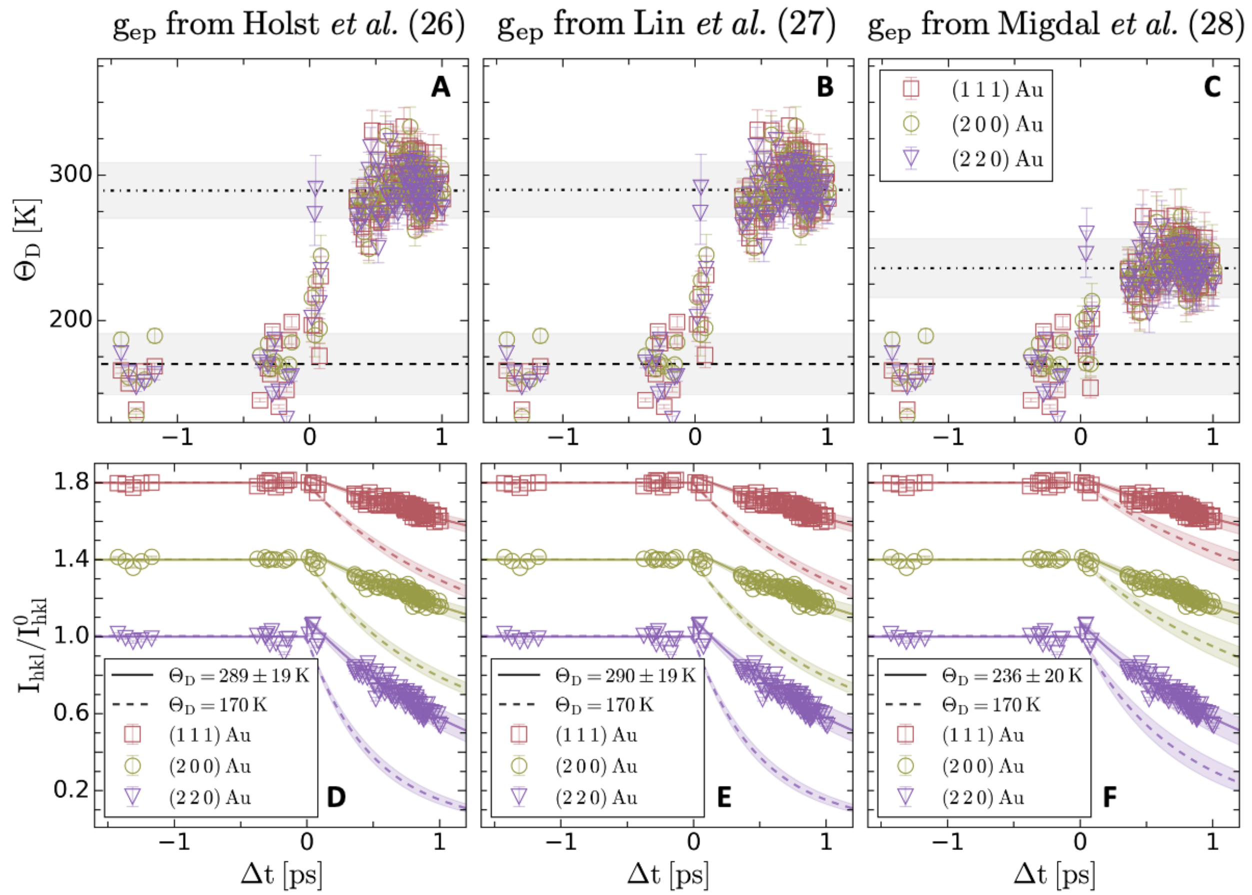

Fig. S3. Temporal evolution of the Debye temperature extracted using the different models. Figure similar to Fig. 2A for the other TTM used in this work.

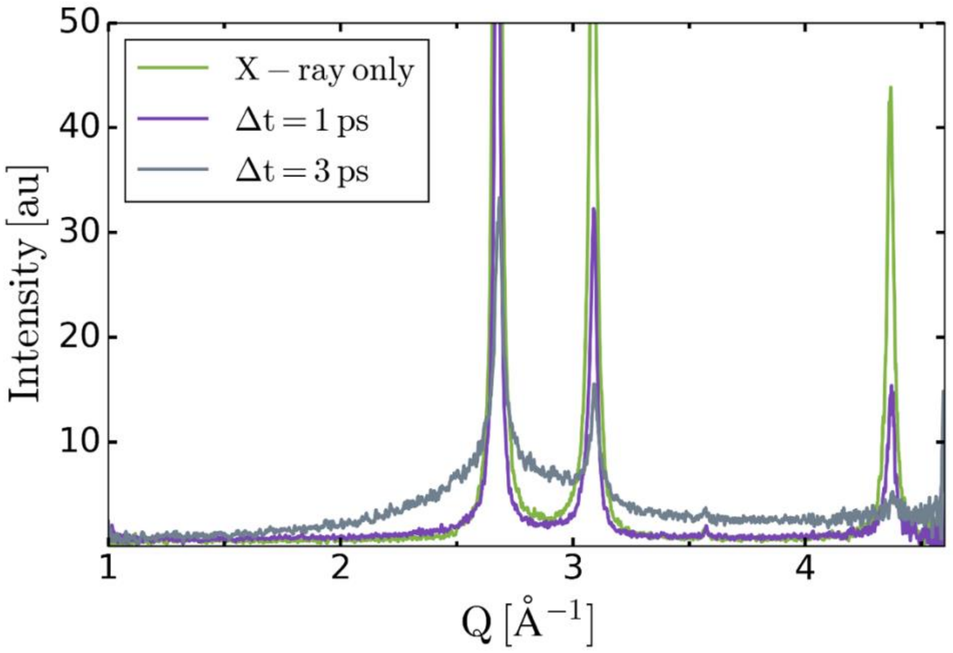

Fig. S4. Single-shot diffraction patterns showing the emergence of liquid scattering.

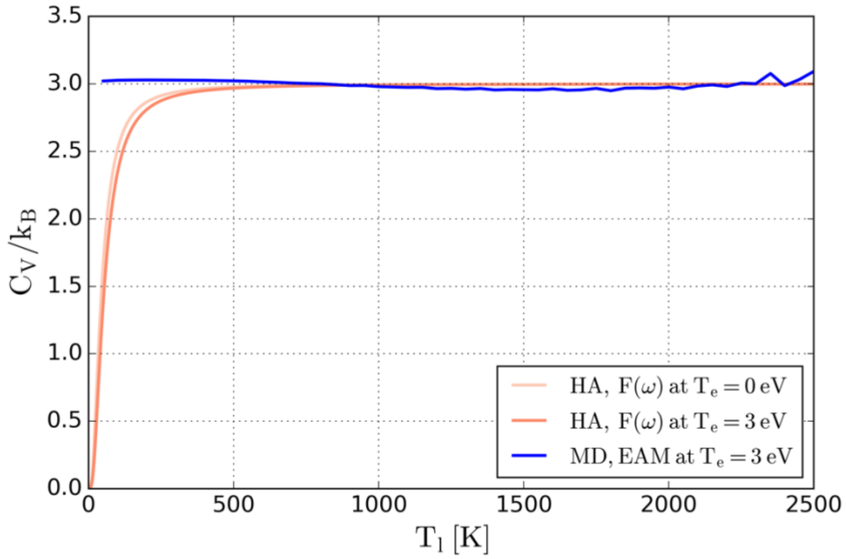

Fig. S5. Evolution of the constant volume heat capacity as a function of the lattice temperature calculated using MD simulations using the interatomic potential from G.E. Norman *et al.* at an electron temperature of 3 eV, close to our experimental conditions.

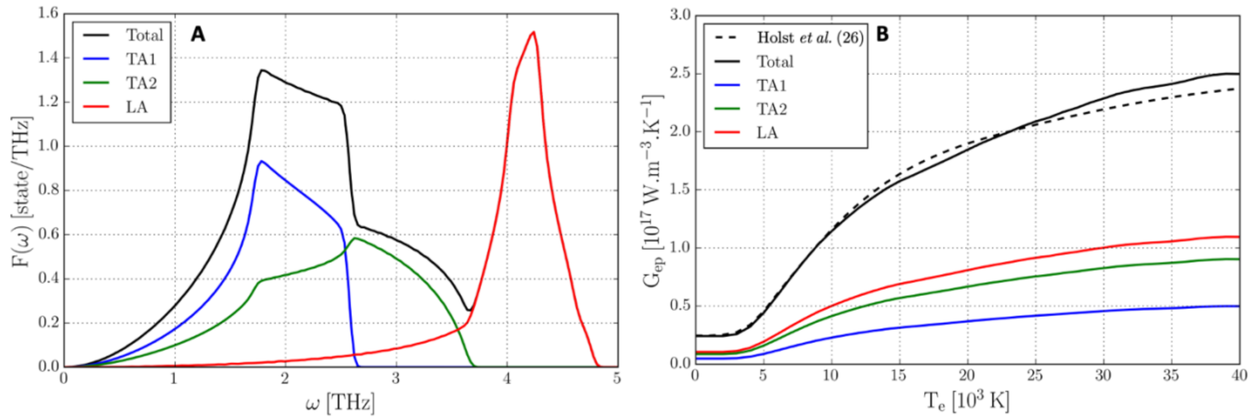

Fig. S6. Total phonon density of states and electron-phonon coupling rate of laser-excited Au (A) Total phonon density of states of Au in black and contribution from each phonon branch. (B) Total electron-phonon coupling rate shown with the solid black line. The contribution for each phonon branch is shown with the color lines. We show the value calculated by Holst *et al.* (26) (black dashed line) for comparison. For the calculation of the mode-dependent electron phonon coupling rates, we used the Eliashberg function calculated at 300 K.

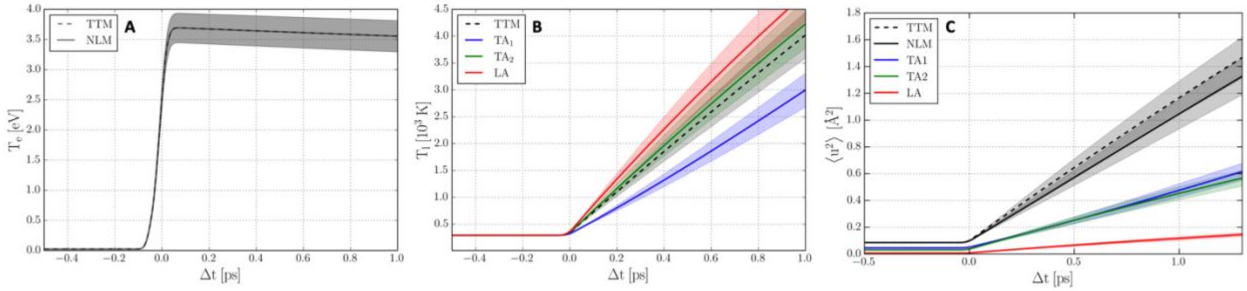

Fig. S7. Comparison between TTM and NLM simulations (A) Evolution of the electron temperature calculated with the TTM (dashed) and the NLM (solid). (B) Evolution of the lattice temperature calculated with the TTM (dashed) and the temperature for each phonon branch calculated with the NLM. (C) Mean square displacement calculated with the TTM (dashed black) and the NLM (solid black). The contribution from each phonon branch is shown with the color lines. For all figures, $G_{pp} = 10^{17} W.m^{-3}.K^{-1}$ and the shaded bands correspond to the 1σ uncertainty on the absorbed energy density.

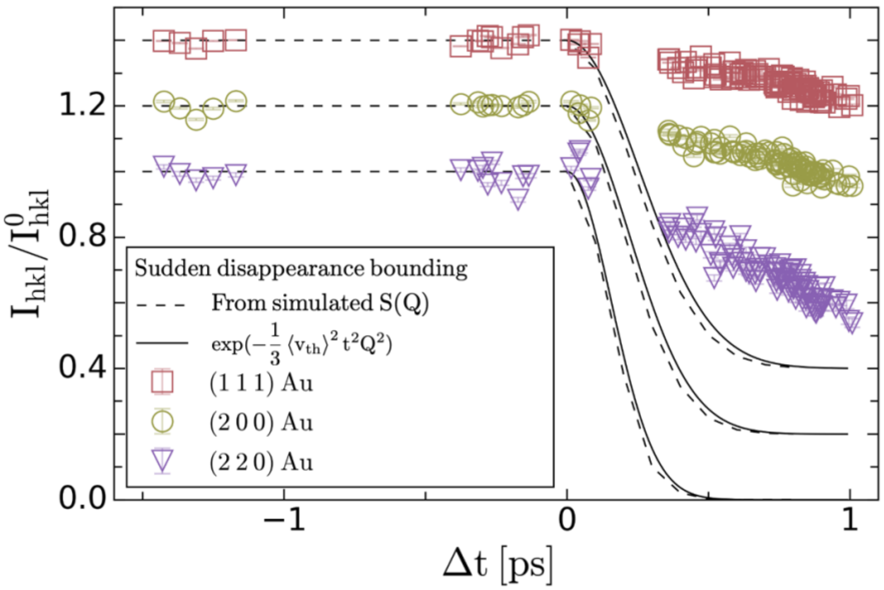

Fig. S8. Evolution of the intensity decay of the (1 1 1), (2 0 0), and (2 2 0) diffraction lines of Au assuming a sudden disappearance of bonding in Au. At 0 ps, the atoms are assumed to follow a ballistic trajectory. The velocity of each atom is extracted from MD simulations performed at 300 K. The dashed lines are calculated from the atomic positions obtained from MD simulations using the static structure factor at t > 0ps and t < 0ps. The solid lines correspond to the expected Debye-Waller decay assuming the atoms follow a ballistic trajectory given by the average thermal velocity as given by Wang *et al.* (54).

REFERENCES

1. C. V. Shank, R. Yen, C. Hirlimann, Time-resolved reflectivity measurements of femtosecond-optical-pulse-induced phase transitions in silicon. *Phys. Rev. Lett.* **50**,454–457 (1983).

2. K. Sokolowski-Tinten, J. Bialkowski, M. Boing, A. Cavalleri, D. von der Linde, Thermal and nonthermal melting of gallium arsenide after femtosecond laser excitation. *Phys. Rev.B* **58**, R11805–R11808 (1998).

3. T. Ao, Y. Ping, K. Widmann, D. F. Price, E. Lee, H. Tam, P. T. Springer, A. Ng, Optical properties in nonequilibrium phase transitions. *Phys. Rev. Lett.* **96**, 055001 (2006).

4. A. Giri, J. T. Gaskins, B. M. Foley, R. Cheaito, P. E. Hopkins, Experimental evidence of excited electron number density and temperature effects on electron-phonon coupling in gold films. *J. Appl. Phys.* **117**, 044305 (2015).

5. Z. Chen, M. Mo, L. Soulard, V. Recoules, P. Hering, Y. Y. Tsui, S. H. Glenzer, A. Ng, Interatomic potential in the nonequilibrium warm dense matter regime. *Phys. Rev. Lett.* **121**, 075002 (2018).

6. Z. Chen, Y. Y. Tsui, M. Z. Mo, R. Fedosejevs, T. Ozaki, V. Recoules, P. A. Sterne, A. Ng, Electron kinetics induced by ultrafast photoexcitation of warm dense matter in a 30-nm-thick foil. *Phys. Rev. Lett.* **127**, 097403 (2021).

7. R. Biswas, V. Ambegaokar, Phonon spectrum of a model of electronically excited silicon. *Phys. Rev. B* **26**, 1980–1988 (1982).

8. V. Recoules, J. Clérouin, G. Zérah, P. M. Anglade, S. Mazevet, Effect of intense laser irradiation on the lattice stability of semiconductors and metals. *Phys. Rev. Lett.* **96**, 055503 (2006).

9. K. Sokolowski-Tinten, C. Blome, J. Blums, A. Cavalleri, C. Dietrich, A. Tarasevitch,I. Uschmann, E. Förster, M. Kammler, M. Horn-von-Hoegen, D. von der Linde, Femtosecond X-ray measurement of coherent lattice vibrations near the Lindemann stability limit. *Nature* **422**, 287–289 (2003).

10. N. A. Smirnov, Copper, gold, and platinum under femtosecond irradiation: Results of first-principles calculations. *Phys. Rev. B* **101**, 094103 (2020).

11. J. Arthur, G. Materlik, R. Tatchyn, H. Winick, The lcls: A fourth generation light source using the slac linac. *Rev. Sci Instrum* **66**, 1987–1989 (1995).

12. H. Weise, W. Decking, Commissioning and first lasing of the European XFEL, in *Proceedings of International Free Electron Laser Conference (FEL'17)* (International Free Electron Laser Conference No. 38, JACoW, 2018), pp. 9–13.

13. L. B. Fletcher, H. J. Lee, T. Döppner, E. Galtier, B. Nagler, P. Heimann, C. Fortmann, S. LePape, T. Ma, M. Millot, A. Pak, D. Turnbull, D. A. Chapman, D. O. Gericke, J. Vorberger, T. White, G. Gregori, M. Wei, B. Barbrel, R. W. Falcone, C. C. Kao, H. Nuhn, J. Welch, U. Zastrau, P. Neumayer, J. B. Hastings, S. H. Glenzer, Ultrabright X-ray laser scattering for dynamic warm dense matter physics. *Nat. Photonics* **9**, 274–279 (2015).

14. R. Li, C. Tang, Y. Du, W. Huang, Q. Du, J. Shi, L. Yan, X. Wang, Experimental demonstration of high quality mev ultrafast electron diffraction. *Rev. Sci. Instrum.* **80**, 083303 (2009).

15. S. P. Weathersby, G. Brown, M. Centurion, T. F. Chase, R. Coffee, J. Corbett, J. P. Eichner, J. C. Frisch, A. R. Fry, M. Gühr, N. Hartmann, C. Hast, R. Hettel, R. K. Jobe, E. N. Jongewaard, J. R. Lewandowski, R. K. Li, A. M. Lindenberg, I. Makasyuk, J. E. May, D. McCormick, M. N. Nguyen, A. H. Reid, X. Shen, K. Sokolowski-Tinten, T. Vecchione, S. L. Vetter, J. Wu, J. Yang,

H. A. Dürr, X. J. Wang, Mega-electron-volt ultrafast electron diffraction at slac national accelerator laboratory. *Rev. Sci. Instrum.* **86**, 073702 (2015).

16. M. Z. Mo, Z. Chen, R. K. Li, M. Dunning, B. B. L. Witte, J. K. Baldwin, L. B. Fletcher, J. B. Kim, A. Ng, R. Redmer, A. H. Reid, P. Shekhar, X. Z. Shen, M. Shen, K. Sokolowski-Tinten, Y. Y. Tsui, Y. Q. Wang, Q. Zheng, X. J. Wang, S. H. Glenzer, Heterogeneous to homogeneous melting transition visualized with ultrafast electron diffraction. *Science* **360**, 1451–1455 (2018).

17. R. Ernstorfer, M. Harb, C. T. Hebeisen, G. Sciaini, T. Dartigalongue, R. J. D. Miller, The formation of warm dense matter: Experimental evidence for electronic bond hardening in gold. *Science* **323**, 1033–1037 (2009).

18. S. I. Anisimov B. Rethfeld, Theory of ultrashort laser pulse interaction with a metal, in *Nonresonant Laser-Matter Interaction (NLMI-9)*, V. I. Konov, M. N. Libenson, Eds. (Society of Photo-Optical Instrumentation Engineers, Conference Series, 1997), vol. 3093, pp. 192–203.

19. S. H. Glenzer, L. B. Fletcher, E. Galtier, B. Nagler, R. Alonso-Mori, B. Barbrel, S. B. Brown, D. A. Chapman, Z. Chen, C. B. Curry, F. Fiuza, E. Gamboa, M. Gauthier, D. O. Gericke, A. Gleason, S. Goede, E. Granados, P. Heimann, J. Kim, D. Kraus, M. J. MacDonald, A. J. Mackinnon, R. Mishra, A. Ravasio, C. Roedel, P. Sperling, W. Schumaker, Y. Y. Tsui, J. Vorberger, U. Zastrau, A. Fry, W. E. White, J. B. Hasting, H. J. Lee, Matter under extreme conditions experiments at the linac coherent light source. *J. Phys. B-At. Mol. Opt. Phys.* **49**, 092001 (2016).

20. E. Cunningham, E. Galtier, G. Dyer, J. Robinson, A. Fry, Pulse contrast enhancement via non-collinear sum-frequency generation with the signal and idler of an optical parametric amplifier. *Appl. Phys. Lett.* **114**, 221106 (2019).

21. W. P. Davey, Precision measurements of the lattice constants of twelve common metals. *Phys. Rev.* **25**, 753–761 (1925).

22. P. Heimann, M. MacDonald, B. Nagler, H. J. Lee, E. Galtier, B. Arnold, Z. Xing, Compound refractive lenses as prefocusing optics for x-ray fel radiation. *J. Synchrotron Rad.* **23**, 425–429 (2016).

23. B. Warren, *X-Ray Diffraction, Dover Books on Physics* (Dover Publications, 2012).

24. M. Yokoo, N. Kawai, K. G. Nakamura, K. Kondo, Y. Tange, T. Tsuchiya, Ultrahigh-pressure scales for gold and platinum at pressures up to 550 GPa. *Phys. Rev. B* **80**, 104114 (2009).

25. M. Mo, Z. Chen, S. Glenzer, Ultrafast visualization of phase transitions in nonequilibrium warm dense matter. *MRS Bull.* **46**, 694–703 (2021).

26. B. Holst, V. Recoules, S. Mazevet, M. Torrent, A. Ng, Z. Chen, S. E. Kirkwood, V. Sametoglu, M. Reid, Y. Y. Tsui, Ab initiomodel of optical properties of two-temperature warm dense matter. *Phys. Rev. B* **90**, 035121 (2014).

27. Z. Lin, L. V. Zhigilei, V. Celli, Electron-phonon coupling and electron heat capacity of metals under conditions of strong electron-phonon nonequilibrium. *Phys. Rev. B* **77**, 075133 (2008).

28. K. P. Migdal, D. K. Il'Nitsky, Y. V. Petrov, N. A. Inogamov, Equations of state, energy transport and two-temperature hydrodynamic simulations for femtosecond laser irradiated copper and gold. *J. Phys. Conf. Ser.* **653**, 012086 (2015).

29. P. B. Allen, Theory of thermal relaxation of electrons in metals. *Phys. Rev. Lett.* **59**, 1460–1463 (1987).

30. M. Torrent, F. Jollet, F. Bottin, G. Zérah, X. Gonze, Implementation of the projector augmented-wave method in the ABINIT code: Application to the study of iron under pressure. *Comput. Mater. Sci.* **42**, 337–351 (2008).

31. X. Gonze, B. Amadon, G. Antonius, F. Arnardi, L. Baguet, J.-M. Beuken, J. Bieder, F. Bottin, J. Bouchet, E. Bousquet, N. Brouwer, F. Bruneval, G. Brunin, T. Cavignac, J.-B. Charraud, W. Chen, M. Côté, S. Cottenier, J. Denier, G. Geneste, P. Ghosez, M. Giantomassi, Y. Gillet, O. Gingras, D. R. Hamann, G. Hautier, X. He, N. Helbig, N. Holzwarth, Y. Jia, F. Jollet, W. Lafargue-Dit-Hauret, K. Lejaeghere, M. A. L. Marques, A. Martin, C. Martins, H. P. C. Miranda, F. Naccarato, K. Persson, G. Petretto, V. Planes, Y. Pouillon, S. Prokhorenko, F. Ricci, G.-M. Rignanese, A. H. Romero, M. M. Schmitt, M. Torrent, M. J. van Setten, B. Van Troeye, M. J. Verstraete, G. Zérah, J. W. Zwanziger, The Abinitproject: Impact, environment and recent developments. *Comput. Phys. Commun.* **248**, 107042 (2020).

32. A. H. Romero, D. C. Allan, B. Amadon, G. Antonius, T. Applencourt, L. Baguet, J. Bieder, F. Bottin, J. Bouchet, E. Bousquet, F. Bruneval, G. Brunin, D. Caliste, M. Côté, J. Denier, C. Dreyer, P. Ghosez, M. Giantomassi, Y. Gillet, O. Gingras, D. R. Hamann, G. Hautier, F. Jollet, G. Jomard, A. Martin, H. P. C. Miranda, F. Naccarato, G. Petretto, N. A. Pike, V. Planes, S. Prokhorenko, T. Rangel, F. Ricci, G.-M. Rignanese, M. Royo, M. Stengel, M. Torrent, M. J. van Setten, B. Van Troeye, M. J. Verstraete, J. Wiktor, J. W. Zwanziger, X. Gonze, ABINIT: Overview and focus on selected capabilities. *J. Chem. Phys.* **152**, 124102 (2020).

33. X. Gonze, F. Jollet, F. Abreu Araujo, D. Adams, B. Amadon, T. Applencourt, C. Audouze, J.-M. Beuken, J. Bieder, A. Bokhanchuk, E. Bousquet, F. Bruneval, D. Caliste, M. Côté, F. Dahm, F. Da Pieve, M. Delaveau, M. Di Gennaro, B. Dorado, C. Espejo, G. Geneste, L. Genovese, A. Gerossier, M. Giantomassi, Y. Gillet, D. R. Hamann, L. He, G. Jomard, J.

Laflamme Janssen, S. Le Roux, A. Levitt, A. Lherbier, F. Liu, I. Lukačević, A. Martin, C. Martins, M. J. T. Oliveira, S. Poncé, Y. Pouillon, T. Rangel, G.-M. Rignanese, A. H. Romero, B. Rousseau, O. Rubel, A. A. Shukri, M. Stankovski, M. Torrent, M. J. Van Setten, B. Van Troeye, M. J. Verstraete, D. Waroquiers, J. Wiktor, B. Xu, A. Zhou, J. W. Zwanziger, Recent developments in the ABINIT software package. *Comput. Phys. Commun.* **205**, 106–131 (2016).

34. F. Jollet, M. Torrent, N. Holzwarth, Generation of projector augmented-wave atomic data: A 71 element validated table in the XML format. *Comput. Phys. Commun.* **185**, 1246–1254 (2014).

35. D. M. Ceperley, B. J. Alder, Ground state of the electron gas by a stochastic method. *Phys. Rev. Lett.* **45**, 566–569 (1980).

36. L. Waldecker, R. Bertoni, J. Vorberger, R. Ernstorfer, Electron-phonon coupling and energy flow in a simple metal beyond the two-temperature approximation. *Phys. Rev. X* **6**, 021003 (2016).

37. P. Maldonado, K. Carva, M. Flammer, P. M. Oppeneer, Theory of out-of-equilibrium ultrafast relaxation dynamics in metals. *Phys. Rev. B* **96**, 174439 (2017).

38. Baron, A. Q. R. High-Resolution Inelastic X-ray Scattering Part II: Scattering Theory, Harmonic Phonons, and Calculations. Cham: Springer International Publishing, (2020) pp. 2213–2250.

39. A. Descamps, B. K. Ofori-Okai, K. Appel, V. Cerantola, A. Comley, J. H. Eggert, L. B. Fletcher, D. O. Gericke, S. Göde, O. Humphries, O. Karnbach, A. Lazicki, R. Loetzsch, D. McGonegle, C. A. J. Palmer, C. Plueckthun, T. R. Preston, R. Redmer, D. G. Senesky, C. Strohm, I. Uschmann, T. G. White, L. Wollenweber, G. Monaco, J. S. Wark, J. B. Hastings, U. Zastrau, G. Gregori, S. H. Glenzer, E. E. McBride, An approach for the measurement of the bulk

temperature of single crystal diamond using an X-ray free electron laser. *Sci. Rep.* **10**, 14564 (2020).

40. E. E. McBride, T. G. White, A. Descamps, L. B. Fletcher, K. Appel, F. P. Condamine, C. B. Curry, F. Dallari, S. Funk, E. Galtier, E. J. Gamboa, M. Gauthier, S. Goede, J. B. Kim, H. J. Lee, B. K. Ofori-Okai, M. Oliver, A. Rigby, C. Schoenwaelder, P. Sun, T. Tschentscher, B. B. L. Witte, U. Zastrau, G. Gregori, B. Nagler, J. Hastings, S. H. Glenzer, G. Monaco, Erratum: "Setup for meV-resolution inelastic X-ray scattering measurements and X-ray diffraction at the Matter in Extreme Conditions Endstation at the Linac Coherent Light Source" [Rev. Sci. Instrum. 89, 10F104 (2018)]. *Rev. Sci. Instrum.* **89**, 10F104 (2018).

41. L. Wollenweber, T. R. Preston, A. Descamps, V. Cerantola, A. Comley, J. H. Eggert, L. B. Fletcher, G. Geloni, D. O. Gericke, S. H. Glenzer, S. Göde, J. Hastings, O. S. Humphries, A. Jenei, O. Karnbach, Z. Konopkova, R. Loetzsch, B. Marx-Glowna, E. E. McBride, D. McGonegle, G. Monaco, B. K. Ofori-Okai, C. A. J. Palmer, C. Plückthun, R. Redmer, C. Strohm, I. Thorpe, T. Tschentscher, I. Uschmann, J. S. Wark, T. G. White, K. Appel, G. Gregori, U. Zastrau, High resolution inelastic x-ray scattering at the high energy density scientific instrument at the European X-Ray Free-Electron Laser. *Rev. Sci. Instrum.* **92**, 013101 (2021).

42. A. Descamps, B. K. Ofori-Okai, J. K. Baldwin, Z. Chen, L. B. Fletcher, S. H. Glenzer, N. J. Hartley, J. B. Hasting, D. Khaghani, M. Mo, B. Nagler, V. Recoules, R. Redmer, M. Schörner, P. Sun, Y. Q. Wang, T. G. White, E. E. McBride, Towards performing high-resolution inelastic X-ray scattering measurements at hard X-ray free-electron lasers coupled with energetic laser drivers. *J. Synchrotron Rad.* **29**, 931–938 (2022).

43. J. Amann, W. Berg, V. Blank, F. J. Decker, Y. Ding, P. Emma, Y. Feng, J. Frisch, D. Fritz, J. Hastings, Z. Huang, J. Krzywinski, R. Lindberg, H. Loos, A. Lutman, H. D. Nuhn, D. Ratner, J.

Rzepiela, D. Shu, Y. Shvyd'ko, S. Spampinati, S. Stoupin, S. Terentyev, E. Trakhtenberg, D. Walz, J. Welch, J. Wu, A. Zholents, D. Zhu, Demonstration of self-seeding in a hard-x-ray free-electron laser. *Nat. Photonics* **6**, 693–698 (2012).

44. G. Blaj, A. Dragone, C. J. Kenney, F. Abu-Nimeh, P. Caragiulo, D. Doering, M. Kwiatkowski, B. Markovic, J. Pines, M. Weaver, S. Boutet, G. Carini, C.-E. Chang, P. Hart, J. Hasi, M. Hayes,R. Herbst, J. Koglin, K. Nakahara, J. Segal, G. Haller, Performance of epix10k, a high dynamic range, gain auto-ranging pixel detector for FELs. *AIP Conf. Proc.* **2054**, 060062(2019).

45. P. Debye, Zur theorie der spezifischen wärmen. *Ann. Phys.* **344**, 789–839 (1912).

46. A.-T. Petit, P.-L. Dulong, Recherches sur quelques points importants de la théorie de la chaleur. *Ann. Chim. Phys.* **10**, 395–413 (1819).

47. D. Ivanov, A. Kuznetsov, V. Lipp, B. Rethfeld, B. Chichkov, M. Garcia, W. Schulz, Short laser pulse nanostructuring of metals: Direct comparison of molecular dynamics modeling and experiment. *Appl. Phys. A* **111**, 675–687 (2013).

48. B. Rethfeld, A. Kaiser, M. Vicanek, G. Simon, Ultrafast dynamics of nonequilibrium electrons in metals under femtosecond laser irradiation. *Phys. Rev. B* **65**, 214303 (2002).

49. A. Suslova, A. Hassanein, Numerical simulation of ballistic electron dynamics and heat transport in metallic targets exposed to ultrashort laser pulse. *J. Appl. Phys.* **124**, 065108 (2018).

50. P. Karna, A. Giri, Effect of intense laser irradiation on the thermal transport properties of metals. *Phys. Rev. B* **107**, 094301 (2023).

51. G. E. Norman, S. V. Starikov, V. V. Stegailov, Atomistic simulation of laser ablation of gold: Effect of pressure relaxation. *J. Exp. Theor. Phys.* **114**, 792–800 (2012).

52. X. Tang, C. W. Li, B. Fultz, Anharmonicity-induced phonon broadening in aluminum at high temperatures. *Phys. Rev. B* **82**, 184301 (2010).

53. X. Wang, J. C. Ekström, A. U. J. Bengtsson, A. Jarnac, A. Jurgilaitis, V.-T. Pham, D. Kroon, H. Enquist, J. Larsson, Role of Thermal Equilibrium Dynamics in Atomic Motion during Nonthermal Laser-Induced Melting. *Phys. Rev. Lett.* **124**, 105701 (2020).

54. E. Fransson, M. Slabanja, P. Erhart, G. Wahnström, Dynasor – A tool for extracting dynamical structure factors and current correlation functions from molecular dynamics simulations. *Adv. Theory Simul.* **4**, 2000240 (2021).

55. C. W. Siders, A. Cavalleri, K. Sokolowski-Tinten, C. Tóth, T. Guo, M. Kammler, M. H. von Hoegen, K. R. Wilson, D. von der Linde, C. P. J. Barty, Detection of nonthermal melting by ultrafast X-ray diffraction. *Science* **286**, 1340–1342 (1999).

56. P. Stampfli, K. H. Bennemanm, Theory for the instability of the diamond structure of Si, Ge, and C induced by a dense electron-hole plasma. *Phys. Rev. B* **42**, 7163–7173(1990).
